# Gas‐Solid Phase Reaction Derived Silver Bismuth Iodide Rudorffite: Structural Insight and Exploring Photocatalytic Potential of CO_2_ Reduction

**DOI:** 10.1002/advs.202309526

**Published:** 2024-04-22

**Authors:** Jia‐Mao Chang, Ting‐Han Lin, Kai‐Chi Hsiao, Kuo‐Ping Chiang, Yin‐Hsuan Chang, Ming‐Chung Wu

**Affiliations:** ^1^ Department of Chemical and Materials Engineering College of Engineering Chang Gung University Taoyuan 33302 Taiwan; ^2^ Center for Sustainability and Energy Technologies Chang Gung University Taoyuan 33302 Taiwan; ^3^ Division of Neonatology Department of Pediatrics Chang Gung Memorial Hospital at Linkou Taoyuan 33305 Taiwan; ^4^ Department of Materials Engineering Ming Chi University of Technology New Taipei City 24301 Taiwan

**Keywords:** CO_2_ photoreduction, crystal structure, delocalized Ag, rudorffite, silver bismuth iodide

## Abstract

Photocatalytic reduction of CO_2_ is a promising strategy to mitigate the effects of global warming by converting CO_2_ into valuable energy‐dense products. Silver bismuth iodide (SBI) is an attractive material owing to its tunable bandgap and favorable band‐edge positions for efficient CO_2_ photoreduction. In this study, SBI materials, including AgBi_2_I_7_, AgBiI_4_, Ag_2_BiI_5_, and Ag_3_BiI_6_ are first synthesized, through gas‐solid reaction by controlling the stoichiometric ratio of reactants. The X‐ray absorption near edge structure (XANES) and extended X‐ray absorption fine structure (EXAFS) results revealed that the distance between Ag‐I is proportional to the degree of Ag ions delocalization, which occupies the vacant sites. That greatly retards the charge recombination at vacant sites. In addition, the surface potential via photo‐assisted Kelvin probe force measurements of various SBI catalysts shows that Ag_3_BiI_6_ exhibits the highest surface potential change due to the rich delocalized Ag ions. This results in effective charge carrier transport and prevention of charge recombination at vacant sites. Taking the above advantages, the averaged CO and CH_4_ production rates for Ag_3_BiI_6_ achieved 0.23 and 0.10 µmol g^−1^ h^−1^, respectively. The findings suggest that Ag_3_BiI_6_ has a high potential as a novel photocatalyst for CO_2_ reduction and sheds light on the possibility of solving environmental contamination and sustainable energy crises.

## Graphical abstract

This study offers valuable structural insights into various silver bismuth iodide (SBI) catalysts and elucidates the relationship between local structure and photocatalytic performance. The presence of delocalized silver ions in silver‐rich SBI catalysts results in the vacant sites occupied, thereby inhibiting charge recombination at these locations and enhancing the photocatalytic performance.

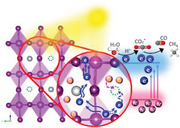

## 1 Introduction

In the last decades, researchers have been searching for a solution to resolve the scenario of global warming and energy shortage. Converting CO_2_, a notorious greenhouse gas, into a carbon‐based fuel through photocatalytic reduction becomes a promising answer to reach the counterbalance between the emission of greenhouse gas and the acquirement of energy.^[^ ^1^, ^2^, ^3^ ^]^ A variety of photocatalysts, such as nitrides, sulfides, phosphides, and metal oxides, have been developed to facilitate this process.^[^ ^4^, ^5^ ^]^ However, a major challenge for efficient CO_2_ conversion is wide band gaps and a short lifetime of photoinduced charges in these materials. This not only limits their visible‐light absorption but also inhibits the generation of electron‐hole pairs for catalytic reactions.^[^ ^6^ ^]^ To overcome these limitations, strategies have been developed, such as finding new materials, enhancing visible‐light absorption, and improving the charge separation properties of photocatalysts. These efforts include the construction of a 2D nanostructure, inducing surface plasmonic effect at a photocatalyst, patterning heterostructures, and doping transition metals in a photocatalyst.^[^ ^7^, ^8^, ^9^, ^10^, ^11^, ^12^, ^13^ ^]^ The goal of these strategies is to develop an efficient, visible‐light‐driven photocatalyst for achieving sustainable CO_2_ conversion and reducing greenhouse gas emissions.^[^ ^14^ ^]^

Among photocatalysts, perovskite and its derivatives, such as rudorffite and double perovskite materials that exhibit relatively low energy bandgap, have raised a surge of application into photocatalytic applications.^[^ ^15^, ^16^ ^]^ Hou et al. first demonstrated the application of colloidal quantum dots cesium lead tribromide perovskites (CsPbBr_3_‐QD), which have tunable optoelectronic properties within the visible spectral region, as photocatalysts for the reduction of CO_2_. Different from organometal halide perovskite materials, the CsPbBr_3_‐QD exhibits astonishing chemical stability in ethyl acetate and water co‐solvents and shows a stable photocatalytic CO_2_ reduction for 12 h.^[^ ^17^ ^]^ However, the soluble lead‐based materials raise a concern about the leakage of lead when applying it to a photocatalytic reaction. Bismuth, holding a stable oxidation state, has emerged as a promising candidate for replacing lead. Ramachandran et al. demonstrated the synthesis of lead‐free Cs_2_AgBiBr_6_ double perovskite nanocrystals via a hot injection method. The Cs_2_AgBiBr_6_ nanocrystals achieved photoreduction of CO_2_ to sustainable solar fuel (CO and CH_4_), achieving a production rate of 14.1 and 9.6 µmol g^−1^ respectively, under AM 1.5 G illumination for 6 h.^[^ ^18^ ^]^ Top‐down synthesis is the other approach to synthesizing Bi‐based perovskite nanocrystals, namely Cs_3_Bi_2_I_9_, Rb_3_Bi_2_I_9_, and MA_3_Bi_2_I_9_. All of them exhibit photocatalytic activity at the gas‐solid interface for the reduction of CO_2_ to CO and CH_4_. Surprisingly, all Bi‐based perovskite derivatives exhibit superior activity to the commercial TiO_2_‐P25 under the same experimental conditions.^[^ ^19^ ^]^

Despite the effective CO_2_ conversion capabilities of double perovskite materials, narrowing the energy bandgap of photocatalysts with appropriate energy levels is always the goal for developing much more effective photocatalysts. The performance of Bi‐based double perovskite photocatalysts is limited by their large bandgap of ≈2.0 eV.^[^ ^20^, ^21^ ^]^ Inheriting from the double perovskite materials, silver bismuth iodide rudorffite materials (SBI) have strong light absorption ability and low toxicity. Their relatively low energy bandgap, being lower than 1.8 eV, allows them to harvest and cover a broader wavelength of incident light.^[^ ^22^, ^23^, ^24^, ^25^, ^26^ ^]^ SBI materials are constructed with two main components, silver iodide (AgI) and bismuth iodide (BiI_3_) octahedrons. By adjusting the stoichiometric ratio of AgI and BiI_3_, a series of SBI materials can be synthesized, including AgBi_2_I_7_, AgBiI_4_, Ag_2_BiI_5_, and Ag_3_BiI_6_. Sharing similar chemical properties of double perovskite photocatalysts, SBI rudorffites also hold good stability in the air compared to conventional organometal halide perovskite materials.^[^ ^27^, ^28^, ^29^, ^30^, ^31^ ^]^ Ramachandran et al. first reported the photocatalytic activity study of Ag_2_BiI_5_ under visible light for methylene blue photodegradation. The Ag_2_BiI_5_ thin film was prepared by thermal evaporation and its photodegradation rate reached 96% after 120 min of illumination.^[^ ^32^ ^]^ Also, the thermal evaporation of SBI is also applied to prepare a freestanding film. The freestanding SBI/cellulose nanofiber composite film demonstrated good hydrophilicity and produced a stable CO_2_ reduction rate within 72 h.^[^ ^33^ ^]^ However, the lack of volume‐confined reaction conditions made it hard to control the stoichiometry and crystal structure of SBI photocatalysts.^[^ ^34^, ^35^ ^]^

Herein, we demonstrate a gas‐solid synthesis reaction in a confined reactor. The targeted crystal structures, including AgBi_2_I_7_, AgBiI_4_, Ag_2_BiI_5_, and Ag_3_BiI_6_, can be regulated by controlling the reactant ratio of Ag/Bi. The confinement of reactants in a closed reactor makes the desired products easy to achieve through the solid‐gas reaction. Among the series of SBI catalysts, Ag_3_BiI_6_ with the silver‐rich composition, exhibits low electron binding energy and a high density of uncoordinated silver. Notably, as the Ag/Bi ratio increases, more delocalized Ag ions occupy the vacant sites, facilitating the charge carrier migration in octahedron‐stacked layer structure and preventing the recombination at vacant sites. The CO_2_ reduction rate, Ag_3_BiI_6_ > Ag_2_BiI_5_ > AgBiI_4_ > AgBi_2_I_7_, shows a significant correlation to the ratio of silver in SBI catalysts. The same tendency of photocatalytic activity and scenario of oxidation state, as well as coordination environments surrounding SBI materials, points out that the metallic Ag in Ag_3_BiI_6_ is the active site for CO_2_ photoreduction. Among these materials, Ag_3_BiI_6_ produced the highest photocatalytic activity, achieving average CO and CH_4_ production rates of 0.23 and 0.10 µmol g^−1^ h^−1^. The results demonstrated that SBI holds substantial potential as an innovative photocatalytic material, offering a promising approach to address challenges related to environmental pollution and sustainable energy production.

## 2 Results and Discussion

**Figure 1:**
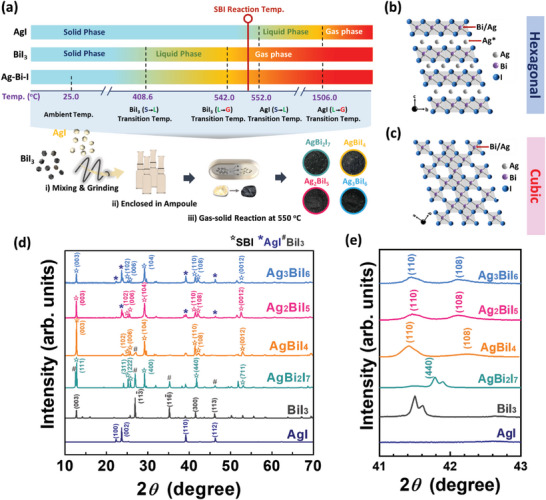
a) Illustration of preparation for SBI catalysts and schematics of b) hexagonal with space group R $\bar{3}$ m and c) cubic structure with space group Fd $\bar{3}$ m of SBI catalysts, and crystal structure analysis of SBI catalysts: d) full XRD diffraction patterns for 2*θ* from 10.0° to 70.0°, e) expanded XRD diffraction patterns for 2*θ* from 41.0° to 43.0°.

SBI catalysts are synthesized using a gas‐solid phase reaction technique, where the composition of the resulting product composition can be manipulated by the stoichiometry of reactants: solid‐phase AgI and gaseous BiI_3_. **Figure** **1**a depicts the procedure of the solid‐gas reaction for SBI catalysts. The raw materials AgI and BiI_3_ crystals were mixed and ground finely and further enclosed into an ampoule. Considering the specific melting and evaporating temperature of AgI and BiI_3_, the reaction temperature of 550 °C was adopted for affordable tolerance, alleviating the slight variation in temperature during the heating process. The various SBI catalysts with distinct crystalline including hexagonal (Figure 1b) and cubic structures (Figure 1c) present black color consistently. To further elucidate the stoichiometry influence of the reactants, the crystal structures of SBI catalysts were investigated by an X‐ray diffractometer (XRD). In Figure 1d, the diffraction peaks at 22.3°, 23.7°, 39.2°, and 46.3° can be ascribed to (100), (002), (110), and (112) planes of hexagonal AgI (PDF # 090374). In addition, the diffraction peaks at 12.8°, 26.9°, 35.3°, 42.1°, and 46.1° are assigned to the (003), (1̅13), (1̅16), (300), and (113) planes of rhombohedral BiI_3_ (PDF # 740457). A series of SBI catalysts are distinctly classified according to previous literature.^[^ ^25^ ^]^ A stable structure of SBI crystal is constructed by two edge‐sharing units of AgI_6_ and BiI_6_ octahedrons. The van der Waals force plays a crucial role in bonding each layer along the c‐axis direction.^[^ ^36^ ^]^ Consequently, the stoichiometric ratio between AgI and BiI_3_ impacts the constructed crystal structures, from cubic Fd $\bar{3}$ m to hexagonal R $\bar{3}$ m.^[^ ^29^ ^]^ In the case of Ag/Bi ratio≥ 1, such as AgBiI_4_, Ag_2_BiI_5_, and Ag_3_BiI_6_, the SBI stacks in a hexagonal R $\bar{3}$ m crystal structure. Also, the presence of the secondary phase of AgI can be simultaneously observed except for the equal stoichiometric composition of AgBiI_4_, which exhibits a single phase. For Ag/Bi ratio < 1, AgBi_2_I_7_, the crystal structure of SBI turns to a cubic structure (Fd $\bar{3}$ m) with another pronounced secondary phase of BiI_3_. Notably, in AgBiI_4_, Ag_2_BiI_5_, and Ag_3_BiI_6_, two diffraction peaks are observed within the 2*θ* range from 41.0° to 43.0°. These peaks correspond to the planes (110) and (108) of hexagonal structure SBI with the space group of R $\bar{3}$ m as shown in Figure 1e. Owing to the relatively low activation energy of Ag migration (0.44 eV), the Ag ions are partially delocalized in the lattice and further occupy the vacant tetrahedral sites between AgI_6_ and BiI_6_ octahedrons with increasing Ag/Bi ratio. The Ag ions from the over‐stoichiometric reactant of AgI replace Bi ions and occupy the Bi central site of the edge‐share octahedron which is illustrated in Figure 1b. On the other hand, the crystal structure goes through a crystal structure evolution. The two peaks at 41.0° and 43.0° merge to one peak at 41.8°, which refers to the plane (440) of the SBI cubic structure with the space group of Fd $\bar{3}$ m, and a shoulder peak. In this case, the Ag central sites of the edge‐share octahedron are occupied by Bi ions. That results in fully eliminating vacancies at tetrahedral sites of octahedrons (Figure 1d). Briefly, the SBI catalysts with a Ag/Bi ratio  ≥ 1 exhibit a hexagonal lattice structure, whereas the bismuth‐rich component with a Ag/Bi ratio < 1, forms a cubic lattice structure. Both structures are composed of cubic close‐packed I‐ion sublattices: AgI and BiI_3_, and the change of Ag/Bi composition results in different crystal structures.

**Figure 2:**
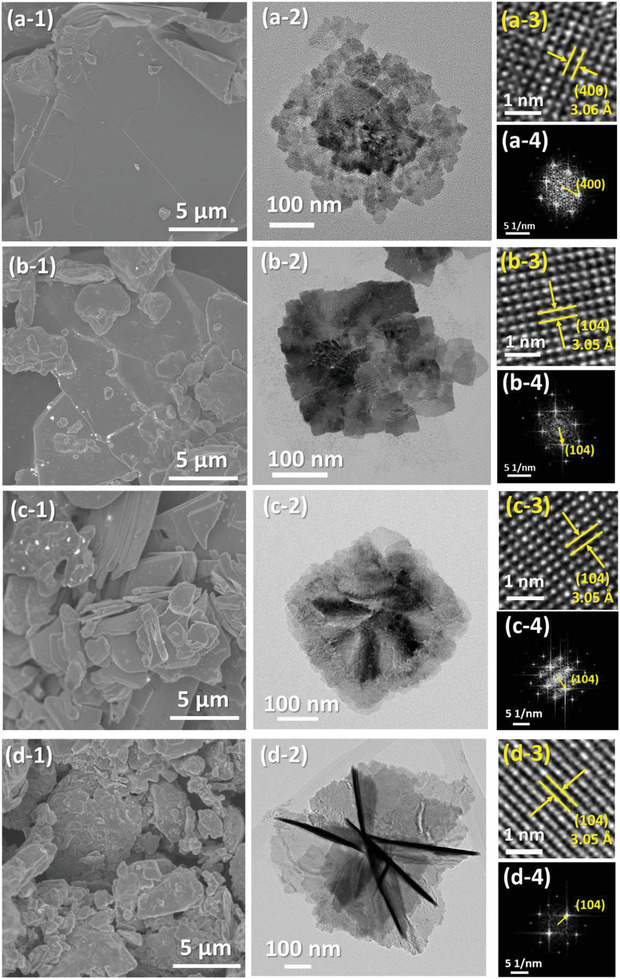
Morphology observation from electron microscopy: (a‐1) Field emission scaning electron microscope images, (a‐2) TEM images, (a‐3) the corresponding HRTEM images, and (a‐4) the corresponding fast Fourier transformed patterns: a) AgBi_2_I_7_, b) AgBiI_4_, c) Ag_2_BiI_5_, and d) Ag_3_BiI_6_.

**Figure 3:**
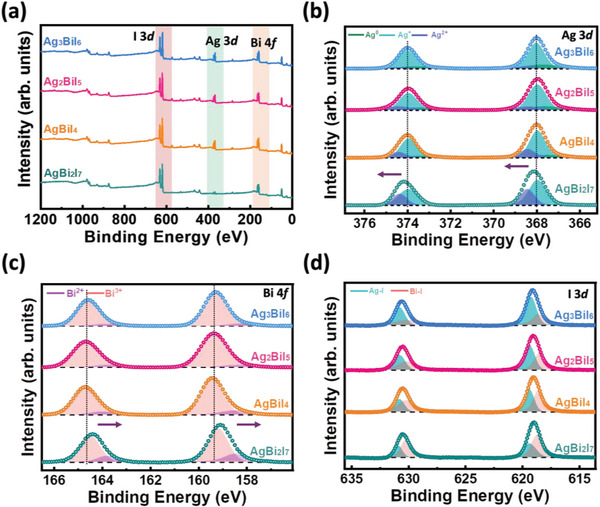
Chemical composition analysis of SBI catalysts from XPS spectra: a) Survey XPS spectra, b) Ag 3*d* orbital, c) Bi 4*f* orbital, and d) I 3*d* orbital.

**Figure 4:**
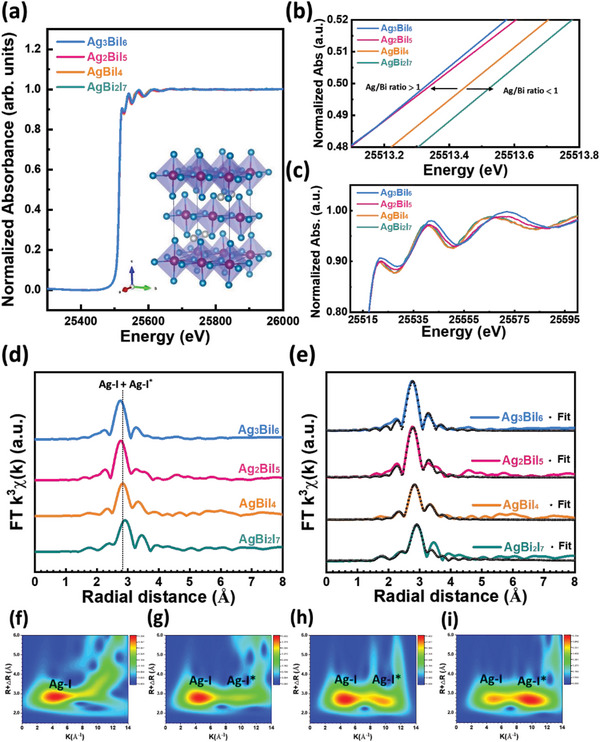
Band edge analysis of various SBI catalysts with XANES: a) Ag K‐edge XANES spectra, b) magnified spectra at normalized absorbance ranged from 0.48 to 0.52, c) magnified spectra at the rising edge d) Fourier transformation of EXAFS spectra in R (reciprocal) space, e) EXAFS fitting curves in R space. Wavelet transform images for f) AgBi_2_I_7_, g) AgBiI_4_, h) Ag_2_BiI_5_, and i) Ag_3_BiI_6_.

The morphology of SBI catalysts is another factor that influences their photocatalytic activity. To investigate the micro‐morphology of SBI catalysts, electron microscopes are used to observe their morphology. **Figure** **2**(a‐1) demonstrates the micro‐morphology of SBI catalysts with various chemical compositions. The bismuth‐rich compositions of AgBi_2_I_7_ are shown in Figure 2(a‐1). The morphology of such a SBI exhibits a larger area of flake crystals. Figure 2(a‐2) shows that the flakes stack together. The high‐resolution transmission electron microscope  (HRTEM) image in Figure 2(a‐3) shows the preferred orientation of the crystal plane (400) with a lattice spacing of 3.06 Å. The corresponding selected area diffraction pattern in Figure 2(a‐4) demonstrates that the crystals of AgBi_2_I_7_ are constructed in a highly ordered way instead of a ring pattern for polycrystalline materials. Additionally, The SBI catalysts with an Ag/Bi ratio≥1, including AgBiI_4_, Ag_2_BiI_5_, and Ag_3_BiI_6_, exhibit a random shape as shown in Figure 2(b‐1),(c‐1),(d‐1). The TEM image shows an intergrown crystallization. The SBI catalysts with am Ag/Bi ratio≥1 grow in a flake‐like shape and merge together as shown in Figure 2(b‐2),(c‐2),(d‐2). The high‐resolution TEM images in Figure 2(b‐3),(c‐3),(d‐3) show the preferred orientation of the crystal plane (104) with a lattice spacing of 3.05 Å, being consistence with the as‐mentioned XRD pattern. The corresponding fast Fourier transformed pattern in Figure 2(b‐4),(c‐4),(d‐4) also demonstrate that the crystals of AgBiI_4_, Ag_2_BiI_5_, and Ag_3_BiI_6_ are constructed in a highly ordered way instead of anisotropic crystallization.

The chemical composition and valence states of SBI catalysts with various compositions are analyzed using X‐ray photoelectron spectroscopy (XPS). **Figure** **3**a demonstrates the survey spectra of SBI catalysts, which reveal the characteristic peaks of Ag 3*d*, Bi 4*f*, and I 3*d*. All of them confirm the presence of these three elements in these SBI catalysts. The binding energy evolution in each element provides information about its neighboring chemical environment. In terms of the photocatalytic activity of SBI catalysts, we speculate that the oxidation states of silver, especially for metallic silver (Ag^0^) and monovalent (Ag^+^), correlate with their photocatalytic activity, and help us determine the condition of delocalized silver ions. Figure 3b shows Ag *3d* orbitals of AgBi_2_I_7_, AgBiI_4_, Ag_2_BiI_5_, and Ag_3_BiI_6_. The binding energies of Ag 3*d_3/2_* and Ag 3*d_5/2_* of AgBiI_4_ are observed at 374.0 and 368.0 eV. It indicates that the Ag^+^ state exists in AgBiI_4_. The calculated compositions of various SBI catalysts are illustrated in Figure S1a (Supporting Information). In the Ag 3*d_3/2_* and Ag 3*d_5/2_* orbitals of AgBiI_4_, the observed peaks can be deconvoluted to Ag^0^ at 373.4 and 367.5 eV, and divalent Ag ions at 374.4 and 368.4 eV, respectively. Table S1 (Supporting Information) summarizes the atomic ratios of different Ag oxidation states in various SBI catalysts. When the Ag/Bi ratio is higher than 1 (Ag_2_BiI_5_ and Ag_3_BiI_6_), the ratio of Ag^+^ and Ag^0^ increases. The Ag^0^ is thought to come from the high electron density surrounding Ag^+^ ions owing to the low density of electron‐attracting species, bismuth ions, in SBI catalysts. Note that Ag^0^ only exists when the stoichiometric ratio of Ag/Bi ≥ 1. It is reported that Ag^+^ ions tend to delocalize within crystal lattice and a portion of them prefers to occupy the vacant tetrahedral sites between AgI_6_ and BiI_6_ octahedrons. It leads to the formation of Ag^0^. The other portions of Ag ions bond with iodide ions in the vacant site and/or the replacement of Bi ion in BiI_6_ octahedrons units, presenting in the form of Ag^+^.

However, when the ratio of Ag/Bi is below 1 (AgBi_2_I_7_), the peaks shift to higher binding energy. It can be attributed to the high electronegativity of the bismuth ions compared to that of the silver ions. Additionally, the abundant bismuth, under this stoichiometric ratio, can lead to a crystal structure transformation, altering the coordination of the silver ions. Owing to the coordination difference, it eventually influences the oxidation state of silver ions. Figure 3c illustrates the Bi 4*f* orbital spectra for the various SBI catalysts. The binding energies of Bi 4*f_5/2_* and Bi 4*f_7/2_* for AgBiI_4_ are observed at 164.6 and 159.3 eV, respectively, being consistent with the valence of Bi^3+^ in SBI. The calculated compositions of different SBIs are illustrated in Figure S1b and Table S2 (Supporting Information), respectively. For Ag/Bi ratio < 1, AgBi_2_I_7_, the binding energy of Bi 4*f* orbital shows a downshift to low binding energy. It is attributed to the high electronegative of Bi ions compared to Ag ions, which attract local electrons of I ions, and further trigger its crystal structure evolution to cubic. Figure 3d illustrates the I 3*d* orbital spectra for various SBI catalysts. These two peaks can be split into Ag‐I and Bi‐I in SBI catalysts according to different binding energies. The relevant split ratio of I 3*d* between Ag‐I and Bi‐I is shown in Figure S1c (Supporting Information) and summarized in Table S3 (Supporting Information). When the Ag/Bi ratio ≥ 1, the composition ratio of Ag‐I increases from 45.1% to 64.2%, while the composition ratio of Bi‐I decreases from 54.9% to 35.8%. When the Ag/Bi ratio < 1, the composition ratio of Ag‐I decreases to 40.2%, while the composition ratio of Bi‐I increases to 59.8%. This scenario comes from the different electronegativity between silver and bismuth ions. Owing to the high electronegativity of bismuth compared to silver, the localized electrons on the orbital of iodide ions become closer to bismuth ions in bismuth‐rich composition. That loosens the bonding between silver and iodide and reduces the electron density around the silver ions.

To gain insight into the microscopic information about each element in SBI catalysts, X‐ray absorption spectroscopy (XAS) is utilized to investigate their electronic structures, valence state variations, and atomic environments with various chemical compositions. **Figure** **4**a demonstrates the X‐ray absorption near edge spectroscopy (XANES) of Ag K‐edge for AgBi_2_I_7_, AgBiI_4_, Ag_2_BiI_5_, and Ag_3_BiI_6_. The detailed XAS spectra for AgI are presented in Figure S2a–d (Supporting Information). To evaluate the oxidation state of Ag in SBI catalysts, the absorption near edge spectra at a normalized absorbance of 0.5 are analyzed, shown in Figure 4b. AgBiI_4_ (Ag/Bi ratio equal to 1) was recognized as a critical composition for crystal phase transition from cubic to hexagonal and is being discussed as a standard reference. With an Ag/Bi ratio > 1, delocalized Ag ions started to occupy the vacant tetrahedral sites and replace the Bi central site in the BiI_6_ octahedral unit. The delocalized Ag ions located at vacancy sites enrich the density of valence electrons and contribute to the significant downshifts of the absorption curve, implying the ratio increment causes Ag ions to change into Ag^0^. On the contrary, when the Ag/Bi ratio is less than 1, Bi ions occupy the central positions of shared edge octahedral, resulting in an upshift of the absorption edge. It is indicative of an increase in the Ag oxidation state. That is, the silver ions prefer to exist in divalent form (Ag^2+^), and consistent with the XPS analysis results. Furthermore, the edge region of XANES presents significant differences for SBI catalysts (Figure 4c). It indicates the change of continuum state and the multiple scattering resonances of low kinetic energy photoelectrons, speculating the distinct fine structure of Ag atoms in various SBI catalysts. Figure S3a,b (Supporting Information) represents the k space and the fitting curves of various SBI catalysts. The k^3^‐weighted extended X‐ray absorption fine structure (EXAFS) curves underwent the Fourier‐transformed (FT) into R space, as illustrated in Figure 4d. The corresponding quantitative least‐squares EXAFS curve, fitted based on the AgBiI_4_ structure (as seen in the inset of Figure 4a), is presented in Figure 4e, offering in‐depth coordination information. In an AgBiI_4_ catalyst, where the Ag/Bi ratio is 1, indicative of a typical AgBiI_4_ structure, the dominant peak in R space reflects two paths associated with the Ag atom due to the octahedral Ag‐I configuration. This suggests Ag ions are tetra‐coordinated with I ions along the a, b‐axes (labeled as Ag‐I) with a distance of ≈2.89 Å. Concurrently, Ag ions are in two‐fold coordination with I ion along with the c‐axis (labeled as Ag‐I^*^), and the corresponding distance is ≈3.09 Å. The detailed bond distances for Ag atoms in various SBI catalysts are outlined in Table S4 (Supporting Information). When the Ag/Bi ratio is lower than 1.0, AgBi_2_I_7_ crystallizes in a cubic crystal structure. Ag ions are hexacoordinated with I ions, and the Ag─I bond lengths across all orientations demonstrate a negligible variation, with a distance of 2.91 Å. In addition, when the Ag/Bi ratio is larger than 1 as in Ag_2_BiI_5_ and Ag_3_BiI_6_, the distances between Ag‐I and Ag‐I^*^ are slightly shortened. For the Ag_2_BiI_5_ catalyst, the distances of Ag‐I and Ag‐I* decreased as the Ag/Bi ratio increased, which is attributed to the Bi atoms being replaced by Ag atoms in the central sites of BiI_6_ octahedral. As Bi ions are replaced by Ag ions, the relatively low oxidation state of Ag ions regulates the attraction force of negative I ions toward the bonded Bi ions. Subsequently, the I ion with a consistent attraction might regulate the attraction again toward the intrinsically bonded Ag, shortening the distance between the Ag and the I. The distances of Ag_2_BiI_5_ for Ag‐I and Ag‐I^*^ are reduced to 3.01 and 2.83 Å, respectively. Identically, with the Ag/Bi ratio increasing further up to Ag_3_BiI_6_, the delocalized Ag ions occupied the vacant sites and dominated the scattering information in the fine structure. The distances of the Ag‐I^*^ path in both the AgI_6_ octahedron and the vacant site are narrowed. To maintain lattice structure, the reduced Ag‐I^*^ path results in the increasing distance of Ag‐I paths for the delocalized Ag ions in the vacant sites. At the same time, by the substantiated results by Wavelet transform (Figure 4f–i), we found that the Ag/Bi ratio increased from 2.0 (Ag_2_BiI_5_) to 3.0 (Ag_3_BiI_6_), with the Ag‐I^*^ domain intensely extended. Incorporating these observations, such as the increased average distance of Ag‐I paths and the expansion of the Ag‐I^*^ domain, it is inferred that the rise in delocalized Ag contributes to the transformation of the SBI crystal and shows a preference for occupying vacant sites.

The electron oxidation state and coordination environment of the Ag in SBI catalysts shows pronounced differences between under and over stoichiometry composition. To realize the synergy effects on Bi in SBI catalysts, Bi L3‐edge XANES of AgBi_2_I_7_, AgBiI_4_, Ag_2_BiI_5_, and Ag_3_BiI_6_ are also conducted and shown in Figures S4 and S5 (Supporting Information). The XAS spectra of pristine BiI_3_ are shown in Figure S6 (Supporting Information) as a reference for the Bi‐I distance. Undoubtedly, the distance between Bi‐I shows a reversed tendency compared to the Ag‐I bonding condition. When the Ag/Bi ratio exceeds 1, an absorption edge upshift is observed, with the main peak in R space comprising two paths. Like the AgI_6_ octahedron, these peaks correspond to 4‐coordinate Bi and I ions along the a, b‐axes (labeled as Bi‐I) at a distance of ≈2.97 Å and 2‐coordinate along the c‐axis (labeled as Bi‐I^*^) at a distance of ≈2.94 Å. The changes in bond lengths among various Ag/Bi ratios are minimal, suggesting that the coordination environment surrounding Bi atoms remains stable during the Ag substitution, even though changes in the electron structure of Bi ions are observed from XANES.

**Figure 5:**
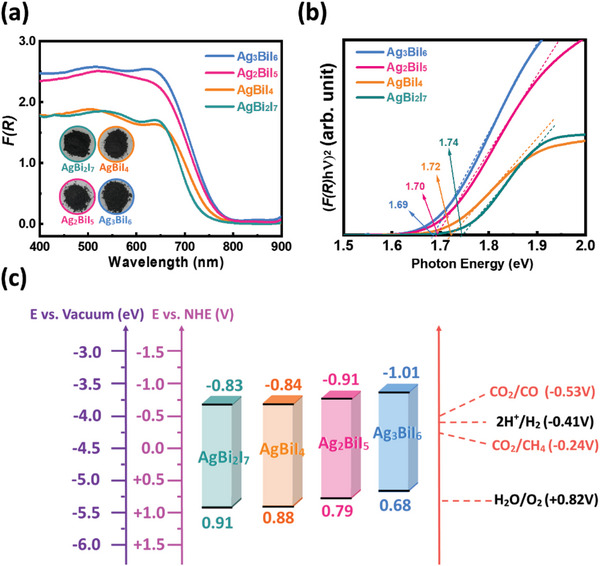
Characterization of energy bandgap and energy level of various SBI catalysts: a) UV–vis DRS spectra, b) corresponding Tauc plot, and c) energy diagram of the VB and CB of various SBI catalysts.

The band gap of SBI catalysts determines their light‐harvesting ability. By ultraviolet‐visible diffuse reflectance spectroscopy (UV–vis DRS), we found all SBI catalysts show strong absorption in the visible region. The absorption increases as the Ag/Bi ratio rises (**Figure** **5**a), which is ascribed to the significant presence of delocalized Ag in SBI catalysts. The excess silver in silver‐rich composition tends to promote their optical properties. At the same time, the Kubelka–Munk equation is applied to calculate the relationship between the photon energy (hν) and (F(R)hν)^2^ and to estimate the bandgap energy *E*_g_ of various SBI catalysts. The calculated band gaps of AgBi_2_I_7_, AgBiI_4_, Ag_2_BiI_5_, and Ag_3_BiI_6_ are 1.74, 1.72, 1.70, and 1.69 eV, respectively (Figure 5b). It is worth noting that the information about band edge positions is crucial to peer and frame a photocatalytic mechanism for a composite photocatalyst. The positions of the conduction band (CB) and valence band (VB) are determined from ultraviolet photoelectron spectroscopy (UPS) and summarized in **Table** **1** and Figures S7 and S8 (Supporting Information). The corresponding energy‐level diagrams are also plotted in Figure 5c. All SBI catalysts exhibit more negative conduction bands than the reduction potential of CO_2_, which can provide a driving force for CO_2_ photoreduction. In addition, the conduction band edge of various SBI catalysts shifted negatively when the concentration of silver increased. It is worth noting that the oxidation states of Ag cations, Bi cations, and I anions contribute differently to forming orbital energy for either the valence band or the conduction band when constructing SBI catalysts. To elucidate the band structure and the elemental orbital contributions to the valence and conduction bands, we further implemented computational simulations utilizing density functional theory (DFT). As illustrated in Figures S9–S11 (Supporting Information), the computed band gaps for SBI catalysts were 1.570 eV for AgBiI_4_, 1.548 eV for Ag_2_BiI_5_, and 1.336 eV for Ag_3_BiI_6_. These decreased values exhibit a consistent trend with the experimental observations. Notably, the valence band contributions were predominantly from the *d* orbitals of Ag and the s orbitals of I, while the *p* orbitals of Bi primarily contributed to the conduction band. This observation aligns with the findings reported in the literature, which indicates that the valence band of SBI catalysts is primarily composed of the 4*d* orbital of silver and the 5*p* orbital of iodine. The 6*p* orbital of bismuth dominates the edge of the conduction band.^[^ ^15^ ^]^ Increasing the Ag composition leads to a higher concentration of delocalized Ag ions, thereby enriching the electron density in the d orbital and further contributing to a noticeable shift in the valence band. Thus, it is proposed that adjusting the ratio of silver to bismuth offers a means to modulate both the energy level and the bandgap of SBI catalysts.

**Table 1:** Detailed energy level of SBI band gap, valence band, and conduction band.

Sample	Band gap [eV]	*E*_VB_ [eV]	*E*_CB_ [eV]
AgBi_2_I_7_	1.74	−5.41	−3.67
AgBiI_4_	1.72	−5.38	−3.66
Ag_2_BiI_5_	1.70	−5.29	−3.59
Ag_3_BiI_6_	1.69	−5.18	−3.49

**Figure 6:**
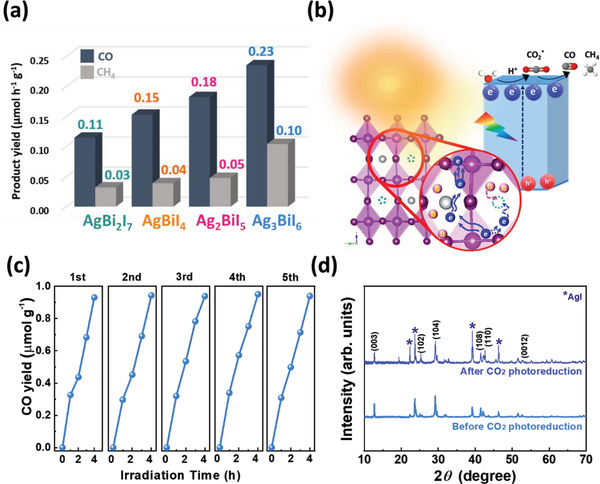
Photocatalytic CO_2_ reduction activities of SBI a) CO and CH_4_ production rate, b) the proposed mechanism of Ag_3_BiI_6_, c) recycling experiments of Ag_3_BiI_6_, and d) XRD pattern before and after photocatalytic CO_2_ reduction.

Although the redox potentials of these SBI catalysts are suitable for photocatalytic CO_2_ reduction from a thermodynamic point of view, the kinetic is also an important factor in evaluating a photocatalyst from a practical point of view. To evaluate the photocatalytic activity of various SBI catalysts, we conducted CO_2_ reduction under a simulated solar light source (AM 1.5). In short, SBI catalysts were confined into a quartz reactor under anaerobic conditions. The reactant of CO_2_ gas was introduced into the reactor. The products were analyzed by gas chromatography (GC). Due to the suitable energy levels of the SBI catalysts, all can facilitate the reduction of CO_2_ to CO or even further to CH_4_ without the need for sacrificial reagents. The results are summarized in **Figures** **6**a and S12 (Supporting Information). Ag_3_BiI_6_ stands out from the series with the highest photocatalytic activity. Its CO_2_ photoreduction rate can achieve 0.23 and 0.10 µmol g^−1^ h^−1^ for CO and CH_4_, respectively. The types of gaseous products were also identified using gas chromatography‐mass spectrometry (GC‐MS), as illustrated in Figure S13 (Supporting Information). The analysis revealed that CO exhibited a mass‐to‐charge ratio (m/z) of 28.1 with a retention time of 5.3 min. CH_4_ displayed m/z values of 16.2, 15.2, and 14.1, corresponding to a retention time of 2.7 min. The control experiment was also carried out as shown in Figure S14 (Supporting Information) to confirm no products which can be produced without photocatalysts. The photocatalytic activity trended lower from Ag_3_BiI_6_ to Ag_2_BiI_5_ to AgBiI_4_ to AgBi_2_I_7_, inferring the activity is highly related to delocalized Ag ions. In general, the vacant sites are occupied by delocalized Ag ions at a high Ag/Bi ratio, which facilitates the charge carrier migration in the c‐axis. It is helpful for the transport from layer structures that are stacked by AgI_6_ and BiI_6_ octahedrons and preventing the recombination at vacant sites, illustrated in Figure 6b. Furthermore, the photoluminescence (PL) spectra and the time‐resolved PL spectra demonstrated that Ag_3_BiI_6_ exhibits lower PL intensity and an extended lifetime, as depicted in Figure S15 and Table S6 (Supporting Information). Additionally, in the CO_2_ adsorption and desorption isotherms, Ag_3_BiI_6_ exhibited the highest adsorption capacities at 298.15 K, illustrated in Figure S16 (Supporting Information). However, increasing the Ag composition to a ratio higher than 75% in the SBI catalyst leads to reduced photocatalytic performance and a tendency to transition from the SBI phase to the AgI phase, as depicted in Figure S17 (Supporting Information). As a result, Ag_3_BiI_6_ exhibits the highest photoreduction activity. Beyond the goal of pursuing a high photocatalytic activity, the durability of photocatalysts is an important factor in evaluating their reliability. Figures 6c and S18 (Supporting Information) reveals that Ag_3_BiI_6_ and other SBI catalysts can maintain their photocatalytic activity even after five iterative cycle tests. Crystal structure analysis in Figure 6d points out that Ag_3_BiI_6_ can maintain the same crystal structure after it was applied to a cycle test.

Besides the photoreduction of CO_2_, the photodegradation of brilliant green, an organic dye, was also conducted to further validate the photocatalytic activity of the as‐prepared SBI photocatalysts, as depicted in Figure S19a (Supporting Information). Without a doubt, the same behavior was also observed in photodegradation. Ag_3_BiI_6_ shows the highest photodegradation activity with a reaction rate constant of 0.0469 min^−1^ as shown in Figure S19b,c (Supporting Information). The reliability test for photodegradation is presented in Figure S19d (Supporting Information), which elucidates that the cycling degradation for organic dye is highly reproduced. The reactive oxygen species test (ROS) helps to gain insight into the mechanism of photocatalytic reaction. Generally, in the ROS test, ammonium oxalate is used for excited holes (h^+^) capturing, tert‐butyl alcohol for hydroxyl radicals (OH∙), and *p*‐benzoquinone for superoxide radicals (∙O_2_^−^), where hydroxyl radicals come from the reaction of hydroxide ions and excited holes and superoxide radicals come from the reaction of surface‐adsorbed oxygen molecules and excited electrons. As shown in Figure S19e (Supporting Information), both degradation tests with scavengers of *p*‐benzoquinone and ammonium oxalate show inferior photodegradation ability to those with tert‐butyl alcohol and no scavengers. That is, the reactive species in the reaction are excited h^+^ and ∙O_2_^−^. The mechanisms of photodegradation are summarized in Figure S19f (Supporting Information). Impressively, after four months of storage, the Ag_3_BiI_6_ photocatalyst still retains the same crystal structure without any degradation or corrosion as shown in Figure S20a (Supporting Information). The photocatalytic activity test in Figure S20b (Supporting Information) shows that the Ag_3_BiI_6_ catalyst can perform the same activity after four weeks of storage in ambient air.

**Figure 7:**
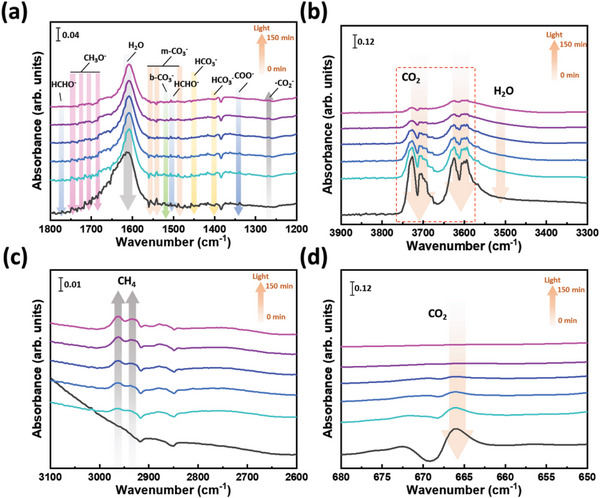
In situ DRIFTS spectra of CO_2_ and H_2_O interaction with Ag_3_BiI_6_: a) at 1200–1800 cm^−1^, b) 3300–3900 m^−1^, c) 2600–3100 cm^−1^ and d) 650–680 cm^−1^ under the light irradiation.

To insight photocatalytic reaction route of SBI catalysts, we carried out the in situ diffuse reflectance infrared Fourier‐transform spectroscopy (DRIFTS) both with and without a halogen lamp, to detect the chemisorption, activation, and mediator transition. **Figures** **7** and S21 (Supporting Information) revealed the surface chemistry of bare Ag_3_BiI_6_ in the dark and under irradiation, providing insights into the compounds adsorbed on the surface. Upon initiating CO_2_ flow into the chamber for 1 h in the dark, various adsorbed species were identified on the surface of the material. These include CO_2_ in δ(CO_2_) at 665 cm^−1^ and ν_3_(CO_2_) at 2341, 2346, as well as in the range of 3550–3750 cm^−1^. Water molecules were detected through δ(OH) at 1609 and ν(OH) at ≈3500 cm^−1^. In addition, the mediators involving bicarbonate (HCO_3_^−^, ν_*as*_(CO_3_) at 1392 and 1455 cm^−1^),^[^ ^37^ ^]^ bidentate carbonate (b‐CO_3_^2−^, ν_*as*_(CO_3_) at 1521 cm^−1^),^[^ ^19^, ^37^ ^]^ monodentate carbonate (m‐CO_3_^2−^, ν_*s*_(CO_3_) at 1483, 1539, and 1552 cm^−1^),^[^ ^19^, ^37^ ^]^ carboxylate (COO^−^, ν_*s*_(CO) at 1337 cm^−1^), methoxide (CH_3_O^−^ in the range of 1685–1745 cm^−1^) and formaldehyde (HCHO^−^ at 1506 and 1769 cm^−1^) were observed. This suggests that CO_2_ and water molecules are effectively adsorbed on the catalyst, leading to the formation of intermediary species. Furthermore, the intensity of these adsorbed species increases as the purge time is extended. Upon initiating irradiation of the catalyst to trigger the CO_2_ photoreduction process, the formation of the •CO_2_^−^ mediator at 1266 cm^−1^ was observed, indicating the activation of CO_2_ reduction through photo‐electron interaction. Moreover, the intensities of CO_2_, H_2_O, HCO_3_^−^, b‐CO_3_^2−^, m‐CO_3_^2−^, COO^−^, CH_3_O^−^, and HCHO^−^ gradually decreased as reaction time increased, implying significant exhaustion of these adsorbed species. This trend suggests that exhaustion prevents the overloading of adsorption on the catalyst surface, which is beneficial for the preservation of active sites. At the same time, an increase in the intensity of methane signals at 2931 and 2961 cm^−1^ was observed, indicating the methane formation during the CO_2_ photoreduction.

**Figure 8:**
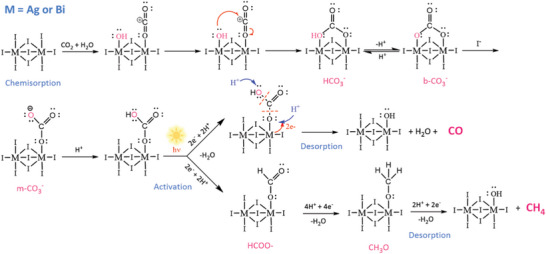
Possible mechanism of CO_2_ molecular adsorption and subsequent photocatalytic reduction pathways to CO and CH_4_ on SBI catalysts.

The possible mechanism for this process is shown in **Figure** **8**. Under dark conditions, the surface MI_6_ (M = Ag, Bi) acts as a Lewis acid, and further interacts with oxygen atoms in CO_2_ and water molecules via chemisorption. The adsorbed water molecules dissociate into H^+^ and OH^−^ and further reacts with CO_2_ to form HCO_3_^−^, b‐CO_3_2^−^, and m‐CO_3_2^−^. Before irradiation, the protonation occurred at m‐CO_3_2^−^ site. As irradiated, two protons are transferred to the protonated m‐CO_3_2^−^ and coupled with photo‐generated electrons, producing HCOO^−^. This intermediate then desorbs H_2_O and CO molecules. A portion HCOO^−^ undergoes the proton coupled electron transfer, resulting in the formation of CH_3_O, which ultimately leads to the desorption of CH_4_.

**Figure 9:**
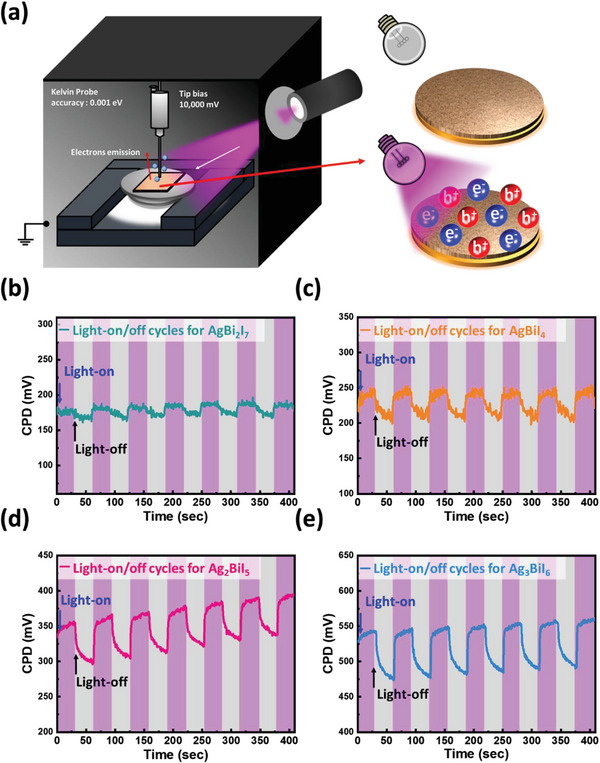
a) The schematic diagram of the photo‐assisted Kelvin probe force analyzer. Contact potential differences with light‐on/off cycles under UV LED (365 nm): b) AgBi_2_I_7_, c) AgBiI_4_, d) Ag_2_BiI_5_, and e) Ag_3_BiI_6_.

Observing the surface potential difference of a photocatalyst under different light sources is a fast and straightforward method to evaluate the photoreduction activity under different light sources. To mimic the surface condition of SBI catalysts during the photocatalytic reaction in ambient conditions, the contact potential difference (CPD), acquired from photo‐assisted Kelvin probe force analyzer, of SBI catalysts are examined under different light irradiation (including ultraviolet (UV) LED at 365 nm, blue LED at 470 nm, green LED at 530 nm, and red LED at 656 nm). The schematics of the photo‐assisted Kelvin probe force analyzer and its charge migration during the illumination are presented in **Figure** **9**a. The on/off illumination not only helps to observe their surface potential evolution but also reveals the relationship between the carrier and photoresponse in SBI catalysts. Herein we recorded the CPD, and the results are shown in Figures 9 and S22–S25 (Supporting Information). The CPD change (ΔCPD) would be defined as follows:

(1) $$\Delta \mathrm{CPD}\;=\;\mathrm{CP}D_{\mathrm{illumination}}\;-\;\mathrm{CP}D_{\mathrm{dark}}$$

The level of CPD change comes from the population of electrons as SBI catalysts are irradiated by a light whose energy is higher than its energy bandgap. The more photogenerated electrons, the more the energy level shifting of the CPD can be observed. Namely, the CPD change is highly correlated with the photoresponse and generation of the electron. Figure 9b–e depicts the shift in CPD  of SBI catalysts when exposed to UV LED irradiation at a wavelength of 365 nm, with comprehensive details provided in **Table** **2**. Notably, among the various SBI catalysts studied, a substantial CPD shift is particularly pronounced in Ag_3_BiI_6_. Furthermore, the CPD changes under different light irradiation conditions for SBI catalysts are presented in Figures S22–S25 (Supporting Information), with detailed information in Table S7 (Supporting Information). It is noteworthy that the CPD shifts observed under blue, green, and red LED irradiation exhibit only minor discrepancies, suggesting that SBI catalysts can populate a similar quantity of electrons under different visible lights with the same intensity. Moreover, an increase in the silver content within SBI catalysts leads to a significantly enhanced CPD change. As mentioned the increase in Ag amount in AgBiI_4_ results in the vacant sites occupied by Ag atoms gradually, which assists in the migration of charge carriers via the channels of the occupied Ag sites. Consequently, the population of electrons is more easily retained on the surface and not recombined. Among various SBI catalysts, Ag_3_BiI_6_ demonstrates the highest surface potential change under all LED illumination, inferring its superior activities in the photodegradation and photoreduction of CO_2_. This suggests that Ag_3_BiI_6_ exhibits a high degree of responsiveness to the entire visible light spectrum, positioning it as a highly promising candidate for use as a visible light photocatalyst.

**Table 2:** Contact potential differences of various SBI catalysts under UV LED illumination.

Sample	*∆*CPD [mV]
AgBi_2_I_7_	12.48 ± 2.26
AgBiI_4_	39.60 ± 3.80
Ag_2_BiI_5_	56.35 ± 3.74
Ag_3_BiI_6_	66.35 ± 2.80

## 3 Conclusion

This study demonstrates the promising potential of SBI materials as a highly efficient photocatalyst for CO_2_ reduction, offering a viable strategy to mitigate the effects of global warming. Through the gas‐solid reaction method, we successfully synthesized various SBI materials, such as AgBi_2_I_7_, AgBiI_4_, Ag_2_BiI_5_, and Ag_3_BiI_6_, by adjusting the stoichiometric ratio of AgI and BiI_3_. Notably, with an Ag/Bi ratio increase, the delocalized Ag ions became rich and occupied the vacant sites. This facilitated the charge carrier migration in an octahedron‐stacked layer structure and prevented the recombination at vacant sites. Hence, the Ag‐rich Ag_3_BiI_6_ catalyst exhibits the highest activity in converting CO_2_ to valuable energy‐dense products, including CO and CH_4_, with average production rates of 0.23 and 0.10 µmol g^−1^ h^−1^, respectively. The Ag_3_BiI_6_ catalyst, with suitable bandgap, visible‐light absorption capacity, favorable band‐edge positions, and long‐term stability, holds great potential for addressing both environmental contamination and the challenges of sustainable energy. Our findings highlight the significance of Ag_3_BiI_6_ as a novel photocatalyst for CO_2_ reduction, paving a way for innovative solutions in tackling climate change and energy‐related issues.

## 4 Experimental Section

### Preparation of Silver Bismuth Iodide Photocatalysts

SBI catalysts with distinct chemical compositions were produced by controlling the molar ratio of silver iodide (Alfa Aesar, 99.999%) and bismuth iodide (Alfa Aesar, 99.999%). Initially, AgI and BiI_3_ were combined in molar ratios of Ag/Bi = 0.5, 1.0, 2.0, and 3.0, followed by thorough grinding. The resulting mixtures were then transferred into ampoules and hermetically sealed. Subsequently, the prepared samples underwent a calcination process at 550 °C for 6 h before being allowed to cool down to room temperature. Upon completion of this process, a series of SBI compositions, AgBi_2_I_7_, AgBiI_4_, Ag_2_BiI_5_, and Ag_3_BiI_6_, were successfully synthesized, manifesting as black powders.

### Characterization of Silver Bismuth Iodide Catalysts

Various SBIs with different chemical compositions were studied using a range of analytical techniques. X‐ray diffraction analysis of crystal structures was performed using a Rigaku SmartLab SE X‐ray diffractometer with Cu *Kα* radiation, with XRD patterns recorded from 2*θ* between 10° and 70° with a 0.01° step at 5.0° min^−1^. Morphology was investigated using a Hitachi SU8010 field‐emission scanning electron microscope at 10.0 kV. Microstructure was observed via a spherical‐aberration Corrected Field Emission Transmission Electron Microscope (JEOL JEM‐ARM200FTH). Chemical composition analysis was performed using X‐ray Photoelectron Spectroscopy with an X‐ray source of Al *Kα* from Thermo Fisher Scientific in the United States of America. X‐ray absorption spectroscopy (XAS) and their extended X‐ray absorption fine structure (EXAFS) were revealed on beamlines 01C1 and 17C1 at the National Synchrotron Radiation Research Center (NSRRC) in Taiwan. UV–vis diffuse reflectance spectra were measured using a JASCO V‐650 UV–vis spectrophotometer in Japan. The Sigma Probe from Thermo VG‐Scientific, equipped with HeI (21.2 eV) light source, was applied to investigate the energy level of various SBIs. The charge transfer dynamics were measured by photoluminescence spectroscopy with a 375 nm diode laser (LDH‐PC‐375, PicoQuant) and time‐resolved photoluminescence spectrum (TR‐PL, UniDRON‐plus, UniNano Tech) at room temperature. For the charge‐decay kinetics calculation, the decay curves were analyzed using a biexponential decay kinetics method. Equations 2 and 3 were employed to perform the biexponential kinetic analysis and determine the average lifetime (*τ_avg_*).

(2) $$F\;\left(t\right)=\;A_{1}\exp\left(-\frac{t}{\tau_{1}}\right)+A_{2}\exp(-\frac{t}{\tau_{2}})$$

(3) $$\tau_{\mathrm{avg}}=\;\frac{\sum_{i}A_{i}\tau_{i}}{\sum_{i}A_{i}}$$

where *A_i_* is the pre‐exponential factor and τ_*i*_ is the decay time.

To investigate the activation route of CO_2_ photoreduction, in situ diffuse reflectance infrared Fourier‐transform spectroscopy (DRIFTS) were recorded in a range of 650 to 4000 cm^−1^ on FTIR spectrometer (Nicolet iS50, Thermo Fisher Scientific) both with and without a halogen lamp (80 W, OSRAM) irradiation. The CO_2_ adsorption/desorption isotherms of the samples were measured using a surface area and porosity analyzer (ASAP2020, Micromeritics Instrument Corporation). The surface potential evaluation with and/or without light irradiation was measured using an ambient scanning Kelvin probe system (SKP5050, KP technology, United Kingdom).

### Computational Simulation

The prediction of the band structure, density of states, and electron density for each element in the samples was conducted using Biovia Materials Studio software. Density functional theory (DFT) calculations employing the Generalized Gradient Approximation Perdew–Burke–Ernzerhof (GGA‐PBE) functional were conducted in CASTEP using a plane‐wave basis set. Norm‐conserving pseudopotentials and reciprocal space representation were adopted for the pseudopotential treatment. The information on models for SBI catalyst are illustrated in Figure S26 and Tables S8–S10 (Supporting Information). After performing geometry optimization on the constructed models, specific parameters were established: an energy cutoff of 350 eV and a k‐point set of 6 × 6 × 7 for AgBiI_4_; an energy cutoff of 400 eV and a k‐point set of 5 × 5 × 3 for Ag_2_BiI_5_; and an energy cutoff of 400 eV with a k‐point set of 9 × 9 × 6 for Ag_3_BiI_6_.

### Photocatalytic Performance of Silver Bismuth Iodide photocatalyst

To evaluate the photocatalytic CO_2_ reduction, all four SBI photocatalysts, AgBi_2_I_7_, AgBiI_4_, Ag_2_BiI_5_, and Ag_3_BiI_6_ were evaluated. In each experiment, 50.0 mg of a photocatalyst was loaded into a quartz reactor with a volume of ≈420.0 ml. The reactor system was purged with N_2_ gas for 30 min to achieve anaerobic conditions. A mixture of CO_2_ and H_2_O vapor was generated by introducing CO_2_ gas into water, and the mixture was fed into the reactor system. Photocatalytic CO_2_ reduction was then conducted under simulated solar illumination (Enlitech, SS‐X, Taiwan) for 8 h. Samples of the gas‐phase product were taken at various time intervals. The reduction products were analyzed using gas chromatography (Shimazhu, Nexis GC‐2030, Japan) to quantify the gaseous products including CO and CH_4_. The mass spectrum of gasous products were indentified by gas chromatography‐mass spectrometry (ISQ7610 GC‐MS, Thermo Fisher Scientific)

## Conflict of Interest

The authors declare no conflict of interest.

## Supporting information

Supporting Information

## Data Availability

Research data are not shared.

## References

[1] E. Gong , S. Ali , C. B. Hiragond , S. H. Kim , S. N. Powar , D. Kim , H. Kim , S.‐I. In , Energy Environ. Sci. 2022, 15, 880.

[2] T. Kong , Y. Jiang , Y. J. Xiong , Chem. Soc. Rev. 2020, 49, 6579. 32789318 10.1039/c9cs00920e

[3] D. Hu , V. V. Ordomsky , A. Y. Khodakov , Appl. Catal. B 2021, 286, 119913.

[4] K. Ren , S. Yue , C. Li , Z. Fang , K. A. Gasem , J. Leszczynski , S. Qu , Z. Wang , M. Fan , J. Mater. Chem. A 2022, 10, 407.

[5] X. Sun , L. Sun , G. Li , Y. Tuo , C. Ye , J. Yang , J. Low , X. Yu , J. H. Bitter , Y. Lei , D. Wang , Y. Li , Angew. Chem., Int. Ed. 2022, 61, e202207677. 10.1002/anie.202207677 35801835

[6] A. J. Cowan , J. R. Durrant , Chem. Soc. Rev. 2013, 42, 2281. 23023269 10.1039/c2cs35305a

[7] C. S. Chen , T. C. Chen , K. L. Chiu , H. C. Wu , C. W. Pao , C. L. Chen , H. C. Hsu , H. M. Kao , Appl. Catal. B 2022, 315, 121596.

[8] L. Liu , Z. Wang , J. Zhang , O. Ruzimuradov , K. Dai , J. Low , Adv. Mater. 2023, 35, 2300643. 10.1002/adma.202300643 36964965

[9] M. Sayed , F. Xu , P. Kuang , J. Low , S. Wang , L. Zhang , J. Yu , Nat. Commun. 2021, 12, 4936. 34400631 10.1038/s41467-021-25007-6 PMC8368040

[10] W. Jiang , H. Loh , B. Q. L. Low , H. Zhu , J. Low , J. Z. X. Heng , K. Y. Tang , Z. Li , X. J. Loh , E. Ye , Y. Xiong , Appl. Catal. B 2023, 321, 122079.

[11] X. Wang , Z. Wang , Y. Li , J. Wang , G. Zhang , Appl. Catal. B 2022, 319, 121895.

[12] L. Wang , B. Zhu , J. Zhang , J. B. Ghasemi , M. Mousavi , J. Yu , Matter 2022, 5, 4187.

[13] C. Hu , X. Chen , J. Low , Y. W. Yang , H. Li , D. Wu , S. Chen , J. Jin , H. Li , H. Ju , C. H. Wang , Z. Lu , R. Long , L. Song , Y. Xiong , Nat. Commun. 2023, 14, 221. 36639386 10.1038/s41467-023-35860-2 PMC9839746

[14] Y. He , Z. Yang , J. Yu , D. Xu , C. Liu , Y. Pan , W. Macyk , F. Xu , J Mater Chem 2023, 11, 14860.

[15] H. C. Sansom , G. F. S. Whitehead , M. S. Dyer , M. Zanella , T. D. Manning , M. J. Pitcher , T. J. Whittles , V. R. Dhanak , J. Alaria , J. B. Claridge , M. J. Rosseinsky , Chem. Mater. 2017, 29, 1538.

[16] Y. F. Mu , J. S. Zhao , L. Y. Wu , K. Y. Tao , Z. L. Liu , F. Q. Bai , D. C. Zhong , M. Zhang , T. B. Lu , Appl. Catal. B 2023, 338, 123024.

[17] J. Hou , S. Cao , Y. Wu , Z. Gao , F. Liang , Y. Sun , Z. Lin , L. Sun , Chemistry 2017, 23, 9481. 28516736 10.1002/chem.201702237

[18] L. Zhou , Y. F. Xu , B. X. Chen , D. B. Kuang , C. Y. Su , Small 2018, 14, 1703762. 10.1002/smll.201703762 29380522

[19] S. S. Bhosale , A. K. Kharade , E. Jokar , A. Fathi , S.‐m Chang , E. W.‐G. Diau , J. Am. Chem. Soc. 2019, 141, 20434. 31800224 10.1021/jacs.9b11089

[20] A. H. Slavney , T. Hu , A. M. Lindenberg , H. I. Karunadasa , J. Am. Chem. Soc. 2016, 138, 2138. 26853379 10.1021/jacs.5b13294

[21] E. T. McClure , M. R. Ball , W. Windl , P. M. Woodward , Chem. Mater. 2016, 28, 1348.

[22] Y. Kim , Z. Yang , A. Jain , O. Voznyy , G. H. Kim , M. Liu , L. N. Quan , F. P. García de Arquer , R. Comin , J. Z. Fan , E. H. Sargent , Angew. Chem., Int. Ed. 2016, 55, 9586. 10.1002/anie.201603608 27355567

[23] A. Bera , S. Paramanik , A. Maiti , A. J. Pal , Phys Rev 2021, 5, 095404.

[24] M. D. Prasad , M. G. Krishna , S. K. Batabyal , ACS Appl. Nano Mater. 2021, 4, 1252.

[25] M. Khazaee , K. Sardashti , C. C. Chung , J. P. Sun , H. Zhou , E. Bergmann , W. A. Dunlap‐Shohl , Q. Han , I. G. Hill , J. Jones , J. Mater. Chem. A 2019, 7, 2095.

[26] M. C. Wu , Q. H. Wang , K. C. Hsiao , S. H. Chen , C. M. Ho , M. H. Jao , Y. H. Chang , W. F. Su , Chem Eng J Adv 2022, 10, 100275.

[27] P. Fourcroy , M. Palazzi , J. Rivet , J. Flahaut , R. Céolin , Mater. Res. Bull. 1979, 14, 325.

[28] T. Oldag , T. Aussieker , H. L. Keller , C. Preitschaft , A. Z. Pfitzner , Z. Anorg. Allg. Chem. 2005, 631, 677.

[29] L. F. Mashadieva , Z. S. Aliev , A. V. Shevelkov , M. B. J. Babanly , Alloy. Compd. 2013, 551, 512.

[30] A. Koedtruad , M. Goto , M. A. Patino , Z. Tan , H. Guo , T. Nakamura , T. Handa , W. T. Chen , Y. C. Chuang , H. S. Sheu , T. Saito , D. Kan , Y. Kanemitsu , A. Wakmiya , Y. Shimakawa , J. Mater. Chem. A 2019, 7, 5583.

[31] Z. Xiao , W. Meng , D. B. Mitzi , Y. Yan , J. Phys. Chem. Lett. 2016, 7, 3903. 27633603 10.1021/acs.jpclett.6b01834

[32] A. A. Ramachandran , B. Krishnan , D. A. Avellaneda , M. I. M. Palma , J. A. A. Martinez , S. Shaji , Surf. Interfaces 2022, 30, 101985.

[33] M. C. Wu , Y. H. Chang , Y. J. Lu , K. C. Hsiao , T. H. Lin , J. M. Chang , K. H. Hsu , J. F. Hsu , K. M. Lee , Mater. Sci. Semicond. Process 2023, 162, 107505.

[34] I. Turkevych , S. Kazaoui , E. Ito , T. Urano , K. Yamada , H. Tomiyasu , H. Yamagishi , M. Kondo , S. ramaki , ChemSusChem 2017, 19, 3754. 10.1002/cssc.201700980 28660660

[35] K. C. Hsiao , Y. F. Yu , C. M. Ho , M. H. Jao , Y. H. Chang , S. H. Chen , Y. H. Chang , W. F. Su , K. M. Lee , M. C. Wu , Chem. Eng. J. 2023, 451, 138807.

[36] A. Crovetto , A. Hajijafarassar , O. Hansen , B. Seger , I. Chorkendorff , P. C. K. Vesborg , Chem. Mater. 2020, 32, 3385.

[37] J. Sheng , Y. He , J. Li , C. Yuan , H. Huang , S. Wang , Y. Sun , Z. Wang , F. Dong , ACS Nano 2020, 14, 13103. 32940453 10.1021/acsnano.0c04659

