# Supplementary material for: Gas‐Solid Phase Reaction Derived Silver Bismuth Iodide Rudorffite: Structural Insight and Exploring Photocatalytic Potential of CO2 Reduction
Source: Adv Sci (Weinh). 2024 Apr 22;11(24):2309526. doi: 10.1002/advs.202309526 (PMC11200016; doi:10.1002/advs.202309526)
Supplement: Supplementary file 1 — Supporting Information [file ADVS-11-2309526-s001.docx]

Supporting Information

Gas-Solid Phase Reaction Derived Silver Bismuth Iodide Rudorffite: Structural Insight and Exploring Photocatalytic Potential of CO_2_ Reduction

Jia-Mao Chang, Ting-Han Lin, Kai-Chi Hsiao, Kuo-Ping Chiang, Yin-Hsuan Chang, Ming-Chung Wu*

**J.-M. Chang, Dr. T.-H. Lin, Dr. K.-C. Hsiao, K.-P. Chiang, Dr. Y.-H. Chang, Prof. M.-C. Wu**

Department of Chemical and Materials Engineering, College of Engineering, Chang Gung University, Taoyuan 33302, Taiwan

E-mail: mingchungwu@cgu.edu.tw (M.-C. Wu)

**Prof. M.-C. Wu**

Center for Sustainability and Energy Technologies, Chang Gung University, Taoyuan 33302, Taiwan

Division of Neonatology, Department of Pediatrics, Chang Gung Memorial Hospital at Linkou, Taoyuan 33305, Taiwan

Department of Materials Engineering, Ming Chi University of Technology, New Taipei City 24301, Taiwan


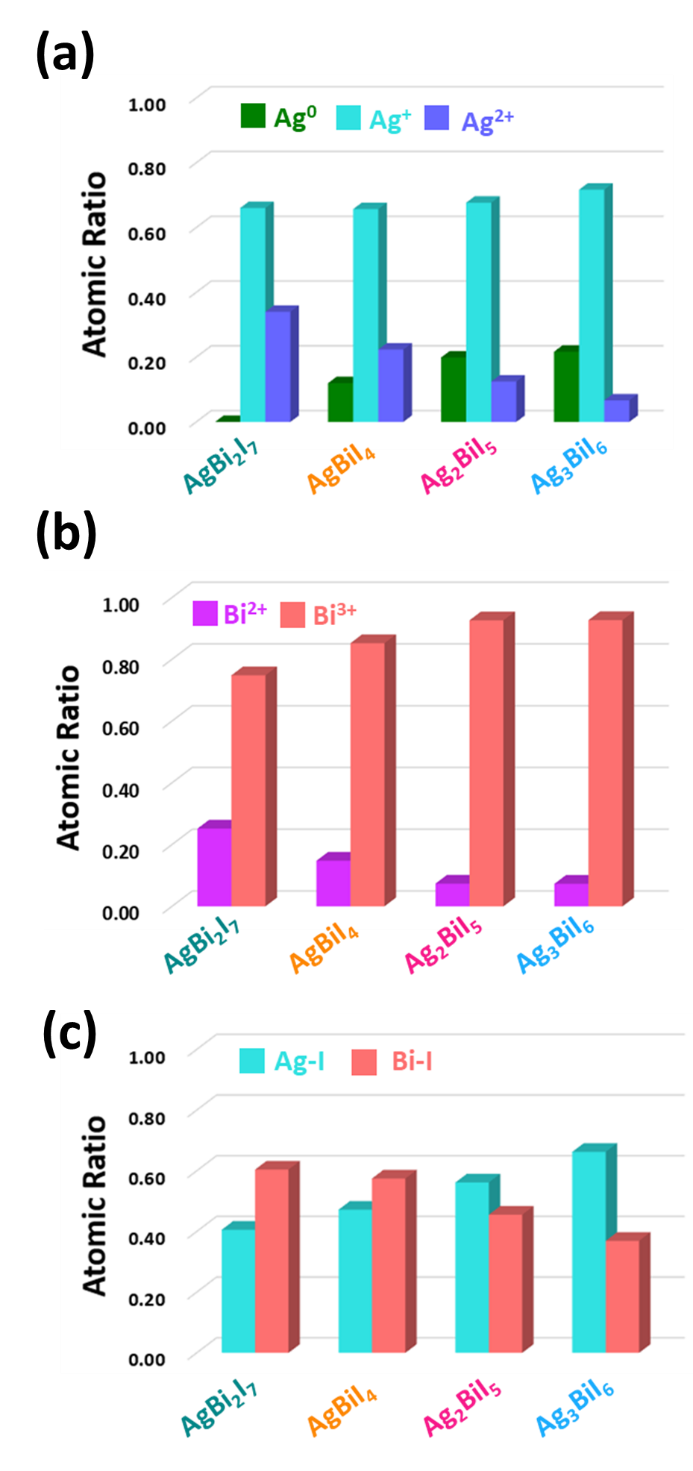


**Figure S1.** Comparison of oxidation state analysis of SBI catalysts, corresponding atomic ratios of **(a)** metallic Ag, monovalent Ag^+^, divalent Ag^2+^ states, **(b)** divalent Bi^2+^, trivalent Bi^3+^ states, **(c)** Ag-I and Bi-I.


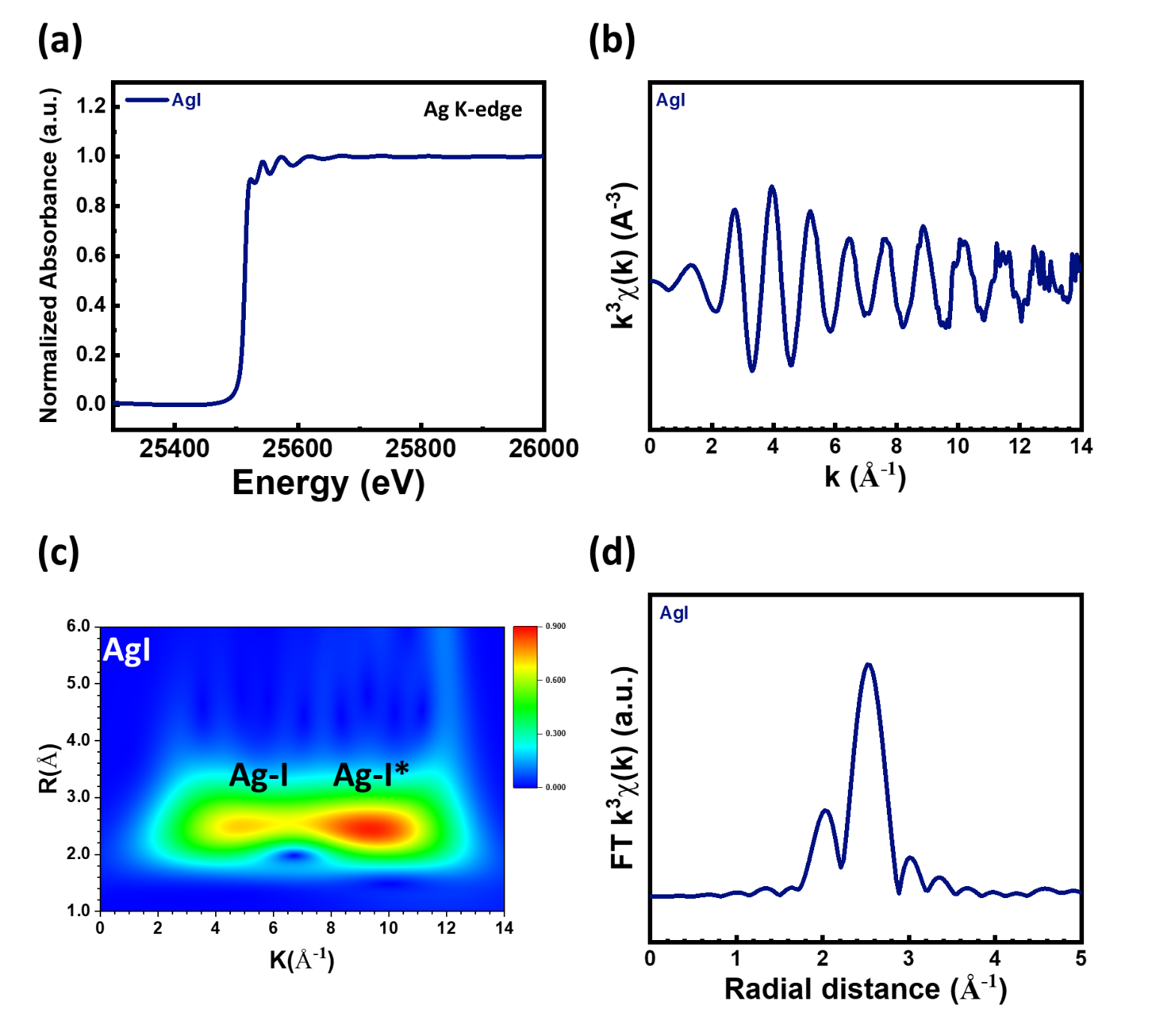


**Figure S2.** Band edge analysis of AgI with X-ray absorption near-edge structures (XANES): **(a)** Ag K-edge XANES spectra, **(b)** k space, **(c)** wavelet transform, and **(d)** Fourier transformation of EXFAS spectra in R (reciprocal) space.


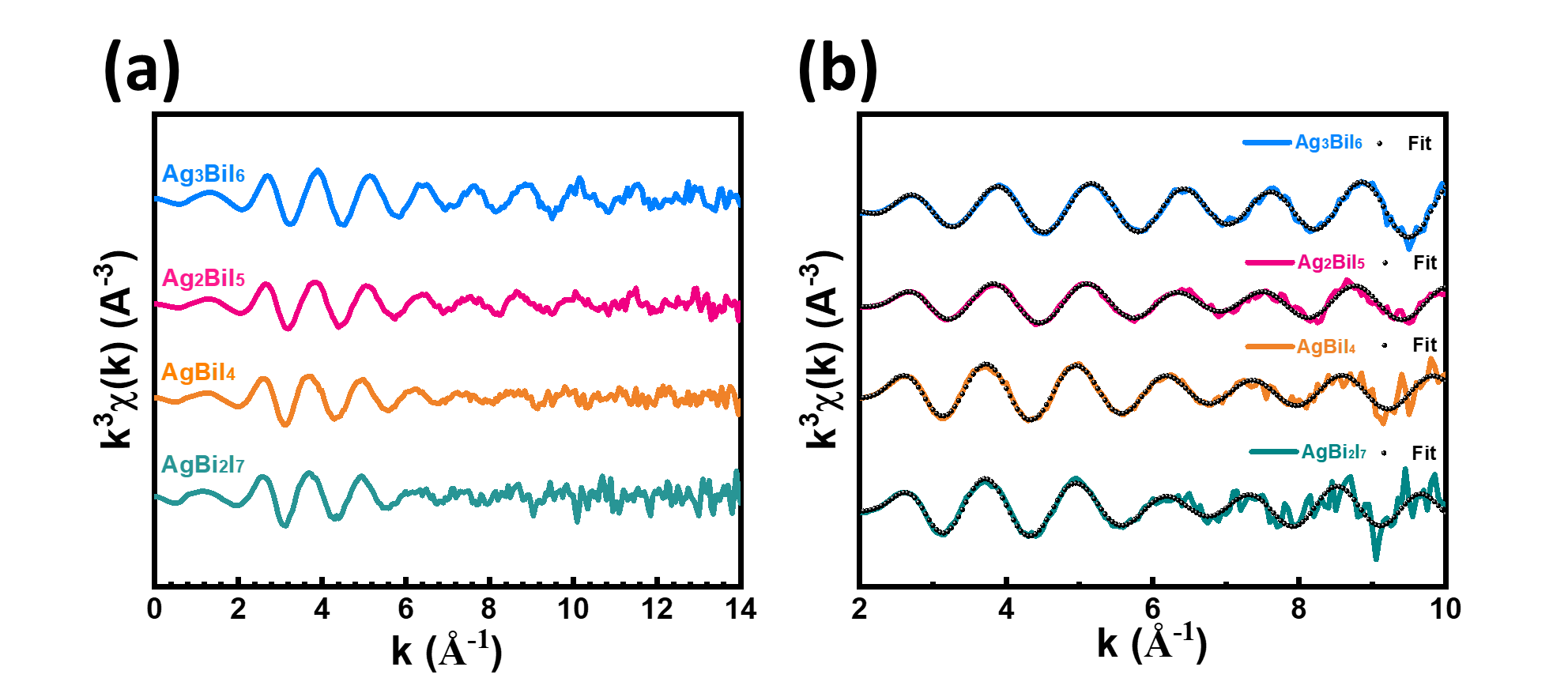


**Figure S3.** Various SBI catalysts with XANES: **(a)** Ag K-edge of k space and **(b)** k^3^-weight Fourier transform spectra fitting curve from Ag K-edge.

.
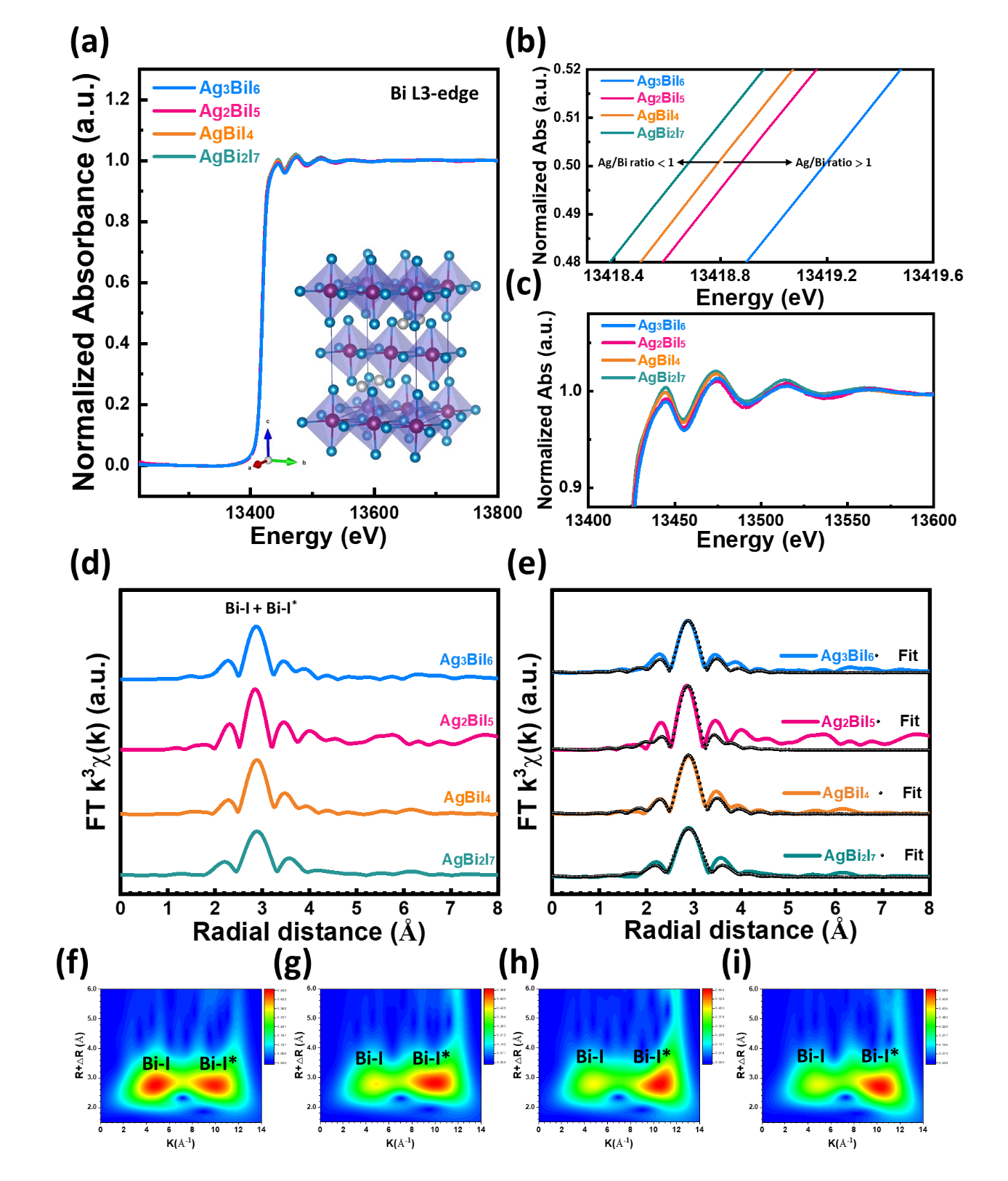


**Figure S4.** Band edge analysis of various SBI catalysts with XANES: **(a)** Bi L3-edge XANES spectra, **(b)** magnified spectra at normalized absorbance ranged from 0.48 to 0.52, **(c)** magnified spectra at the rising edge. **(d)** Fourier transformation of EXAFS spectra in R (reciprocal) space, **(e)** EXAFS fitting curves in R space. Wavelet transform images for **(f)** AgBi_2_I_7_, **(g)** AgBiI_4_, **(h)** Ag_2_BiI_5_, and **(i)** Ag_3_BiI_6_.


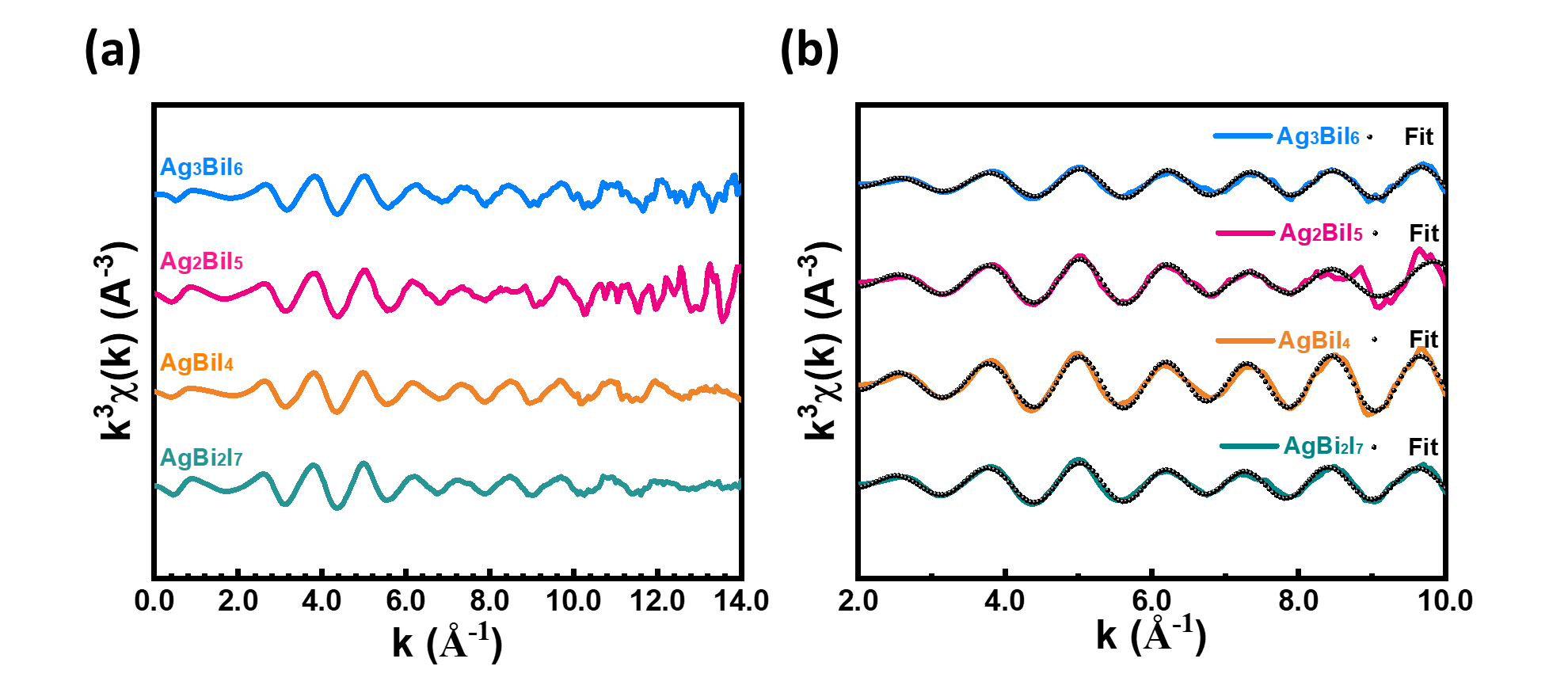


**Figure S5.** Various SBI catalysts with XANES: **(a)** Bi L3-edge of k space and **(b)** k^3^-weight Fourier transform spectra fitting curve from Bi L3-edge.

**
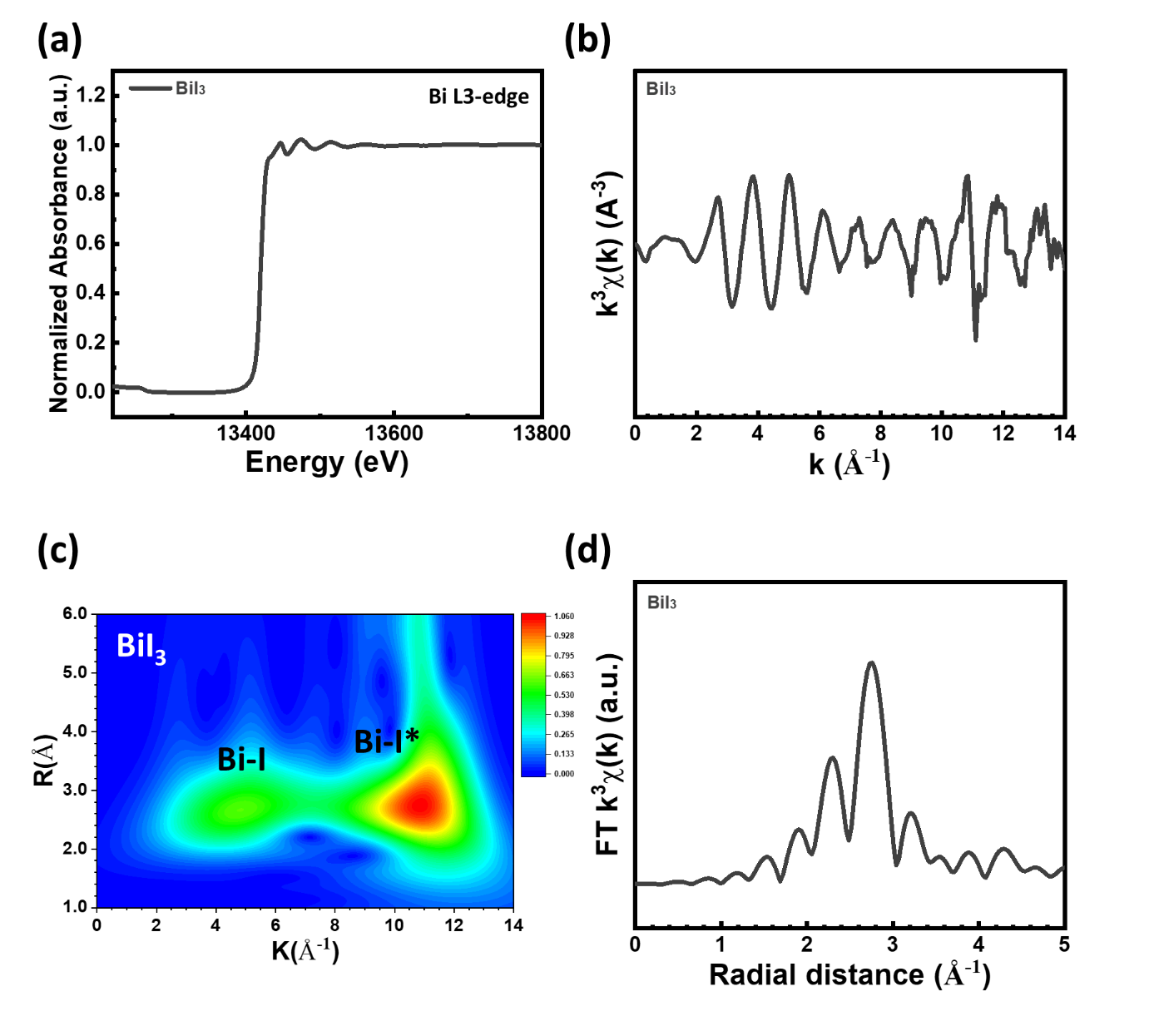
**

**Figure S6.** Band edge analysis of BiI_3_ with XANES: **(a)** Bi L3-edge XANES spectra, **(b)** k space, **(c)** wavelet transform, and **(d)** Fourier transformation of EXFAS spectra in R (reciprocal) space.


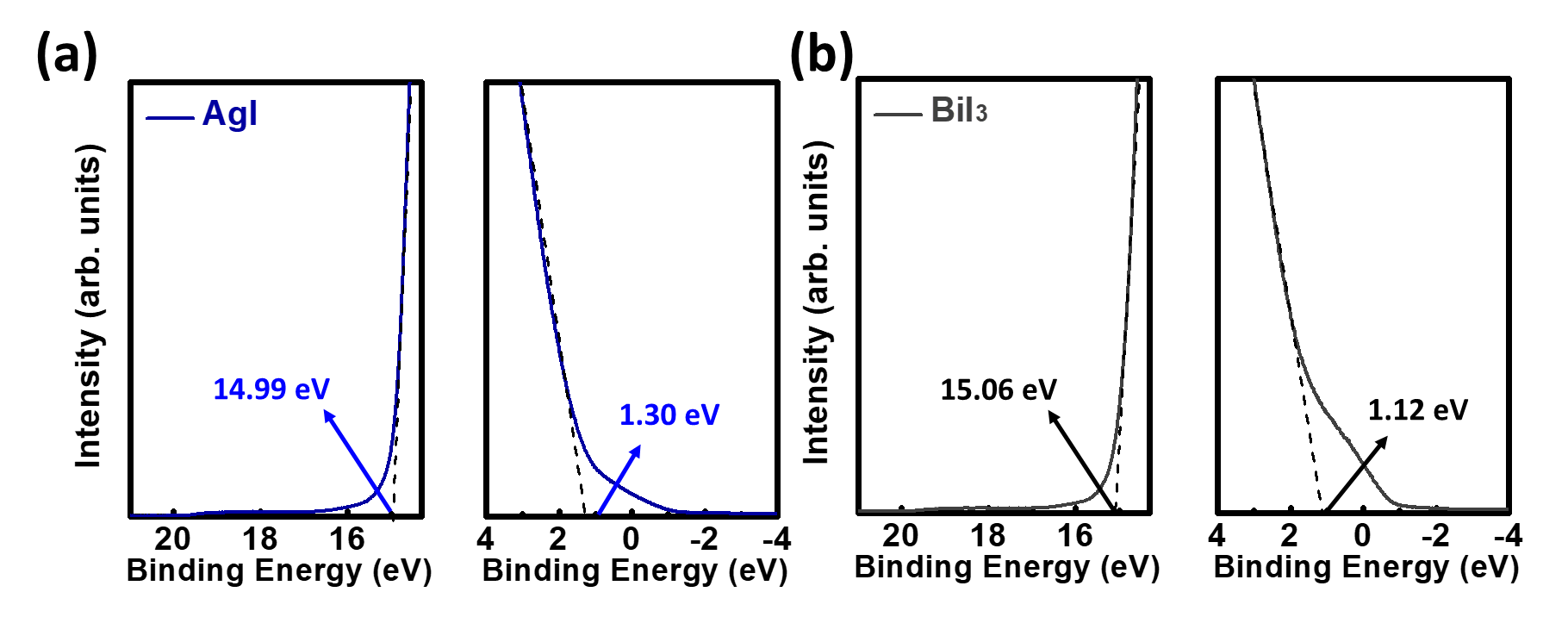


**Figure S7.** Band structure characterization from UPS spectra :**(a)** AgI, and **(b)** BiI_3_.


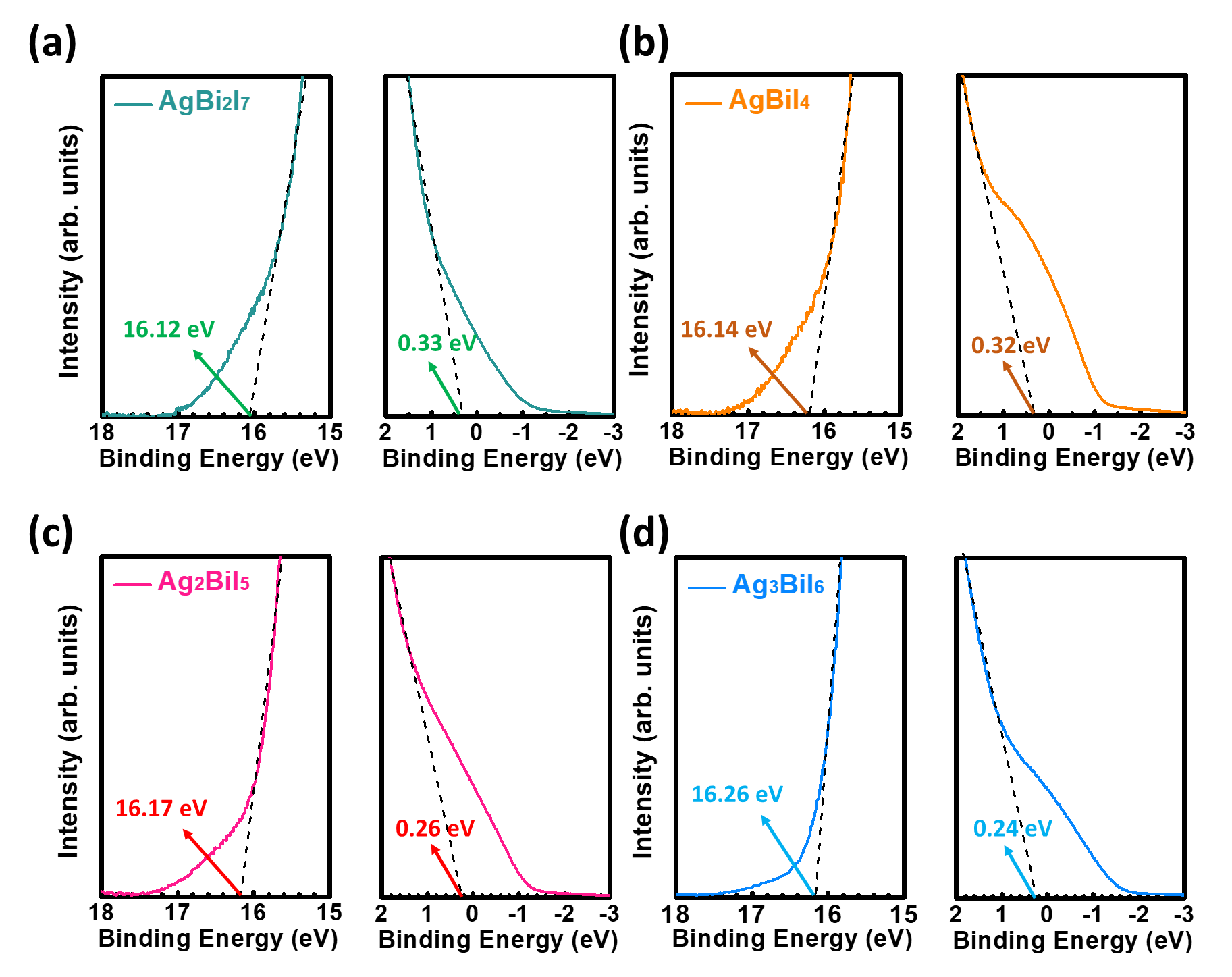


**Figure S8.** Band structure characterization from UPS spectra :**(a)** AgBi_2_I_7_, **(b)** AgBiI_4_, **(c)** Ag_2_BiI_5_, and **(d)** Ag_3_BiI_6_.

**
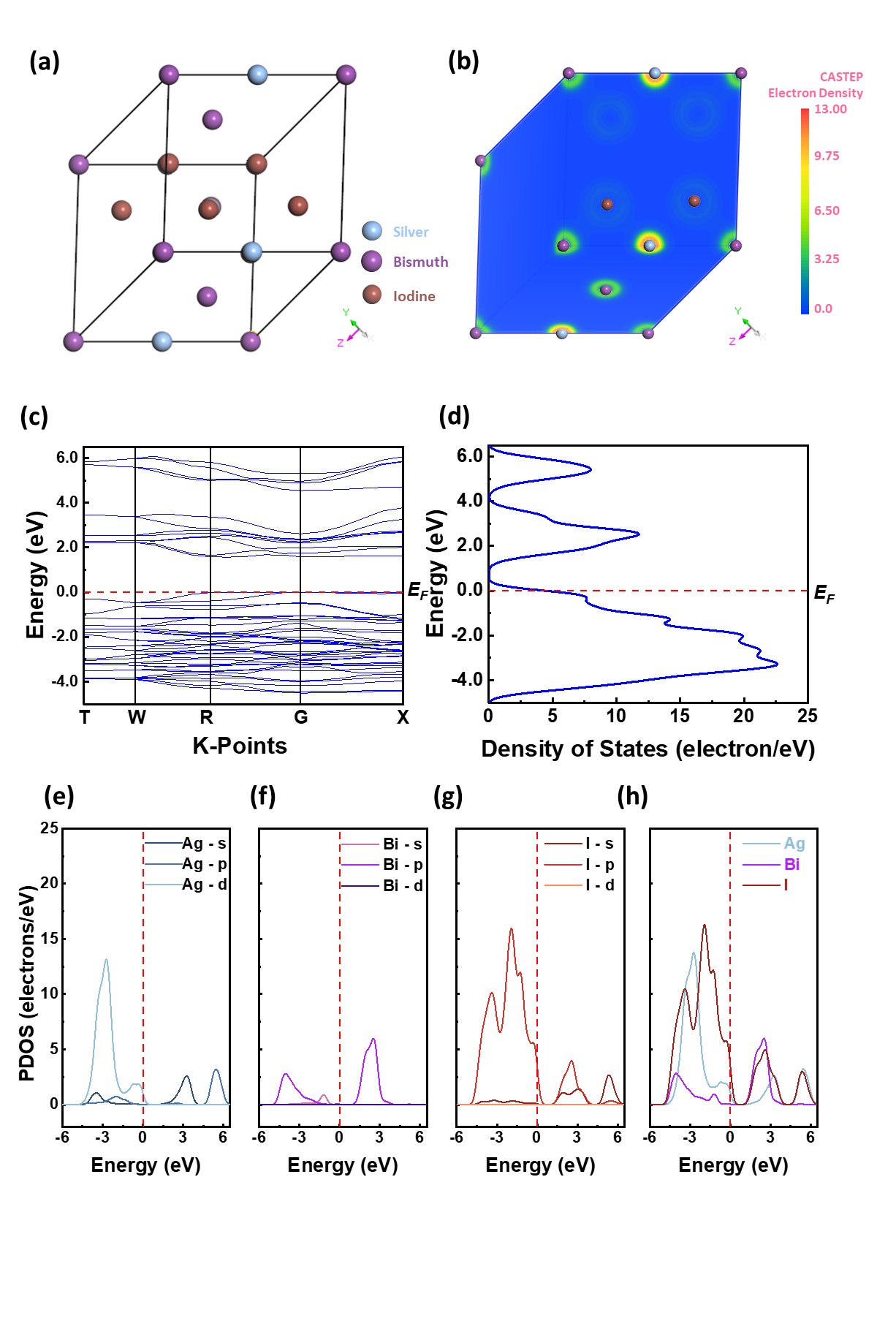
**

**Figure S9.** DFT-based computational analysis of AgBiI_4_. **(a)** Optimized primitive cell structure, **(b)** electron density map, **(c)** band structure, **(d)** total density of state (DOS), **(e)-(g)** Partial DOS for Ag, Bi, and I orbitals, detailing the contribution of each atomic species to the electronic structure, and **(h)** projected DOS of each element delineating the energy statescontribution to the valence and conduction bands.

**
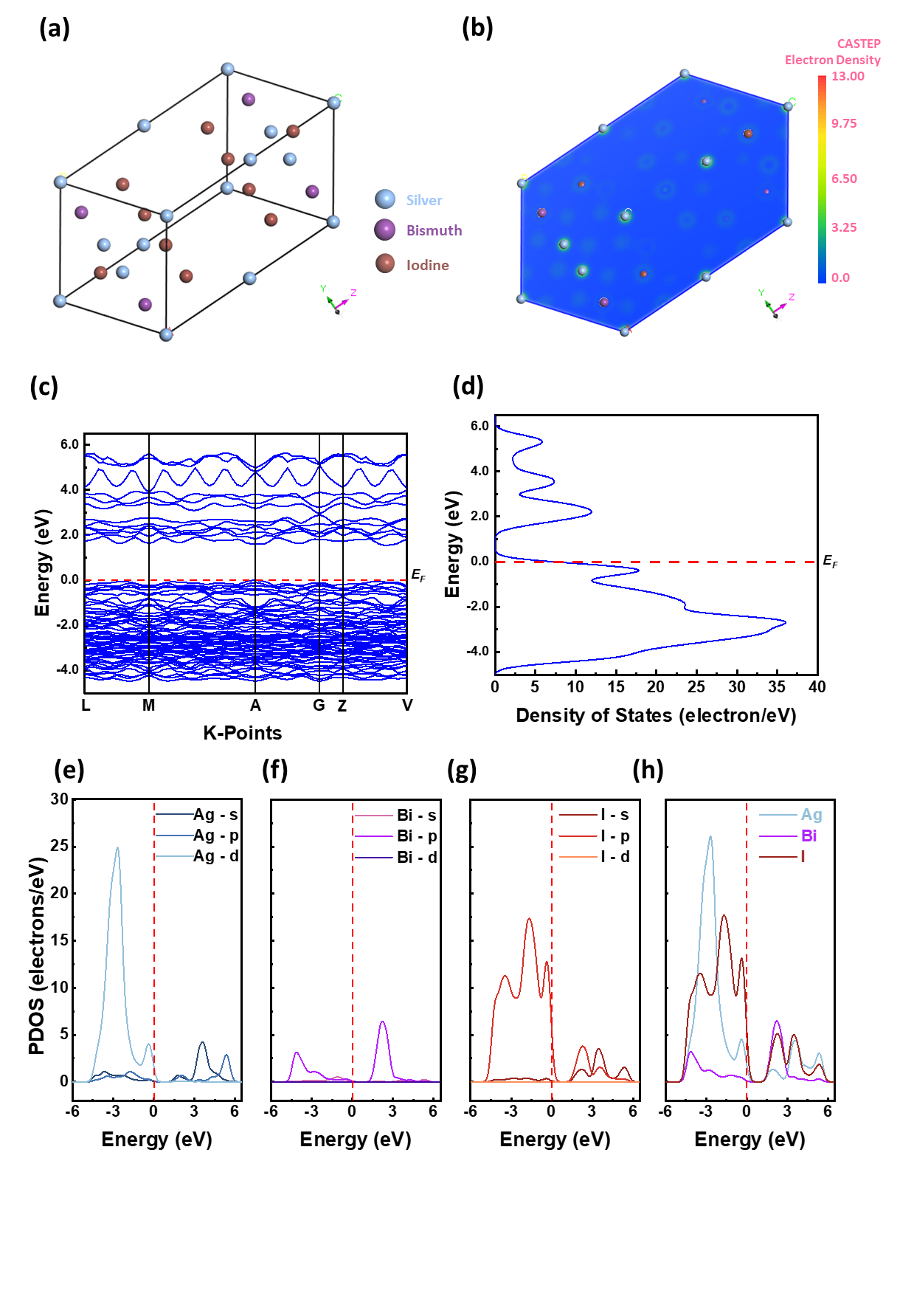
**

**Figure S10.** DFT-based computational analysis of Ag_2_BiI_5_. **(a)** Optimized primitive cell structure, **(b)** electron density map, **(c)** band structure, **(d)** total density of state (DOS), **(e)-(g)** Partial DOS for Ag, Bi, and I orbitals, detailing the contribution of each atomic species to the electronic structure, and **(h)** projected DOS of each element delineating the energy statescontribution to the valence and conduction bands.

**
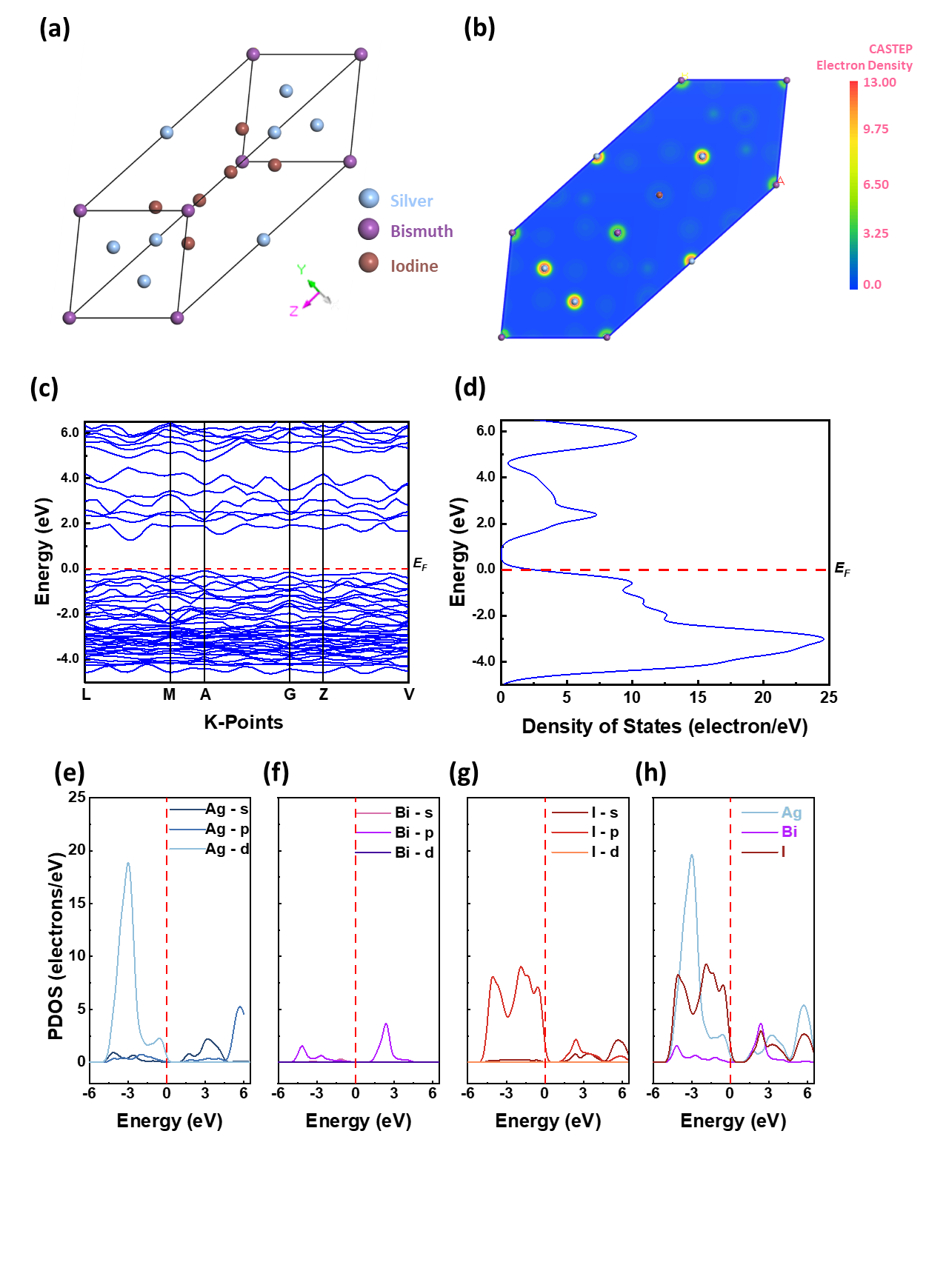
**

**Figure S11.** DFT-based computational analysis of Ag_3_BiI_6_. **(a)** Optimized primitive cell structure, **(b)** electron density map, **(c)** band structure, **(d)** total density of state (DOS), **(e)-(g)** Partial DOS for Ag, Bi, and I orbitals, detailing the contribution of each atomic species to the electronic structure, and **(h)** projected DOS of each element delineating the energy statescontribution to the valence and conduction bands.

**
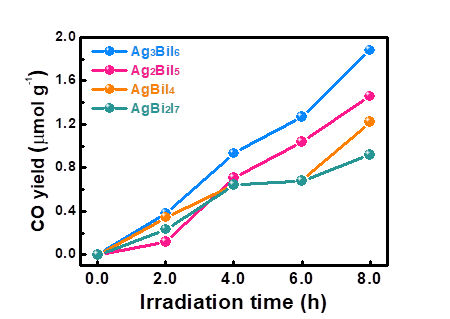
**

**Figure S12.** Photocatalytic CO_2_ reduction activities of SBI for 8-h irradiation.


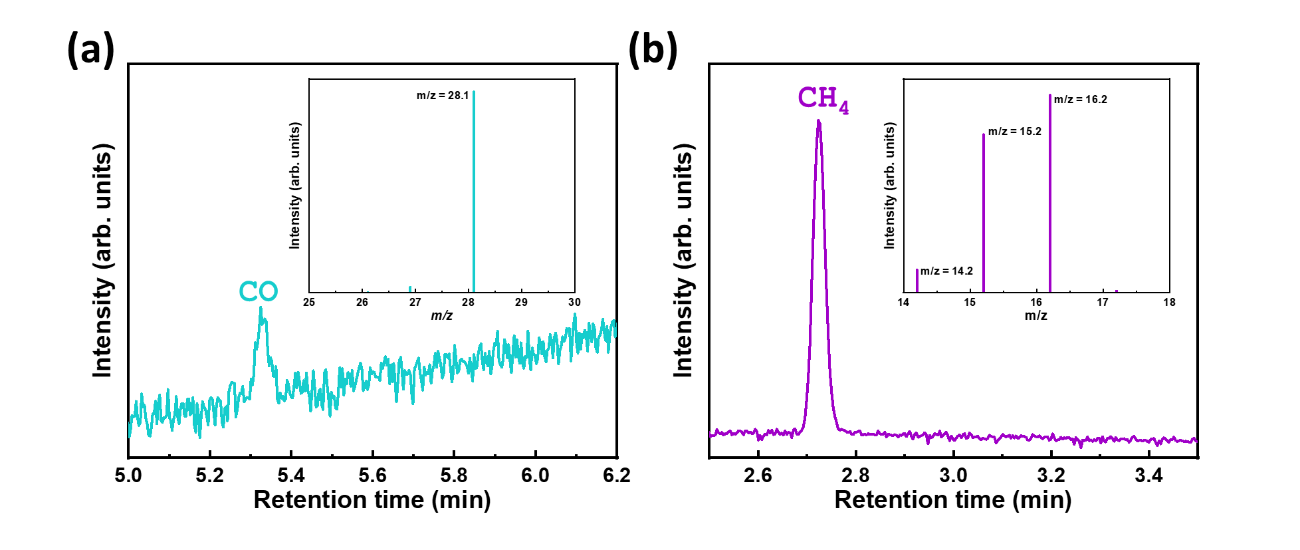


**Figure S13**. Mass Spectra of Gaseous Products from CO_2_ Photoreduction. **(a)** CO and **(b)** CH_4_.

**
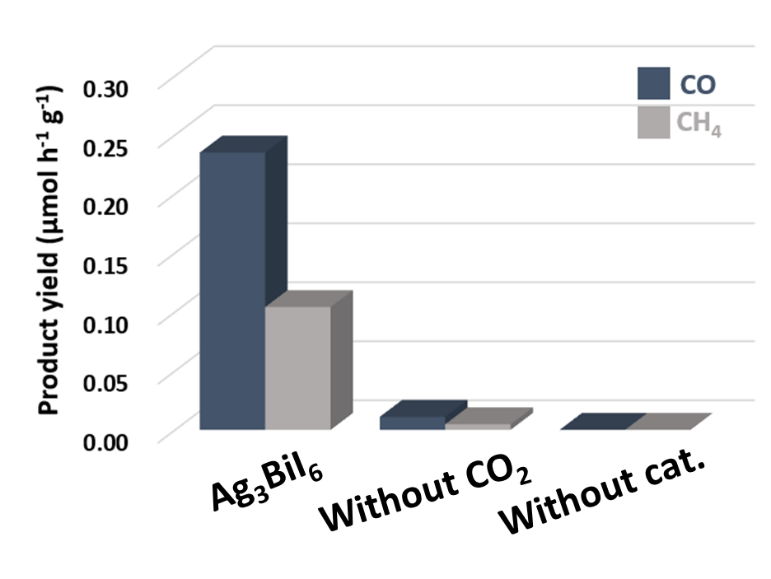
**

**Figure S14.** Control experiments.


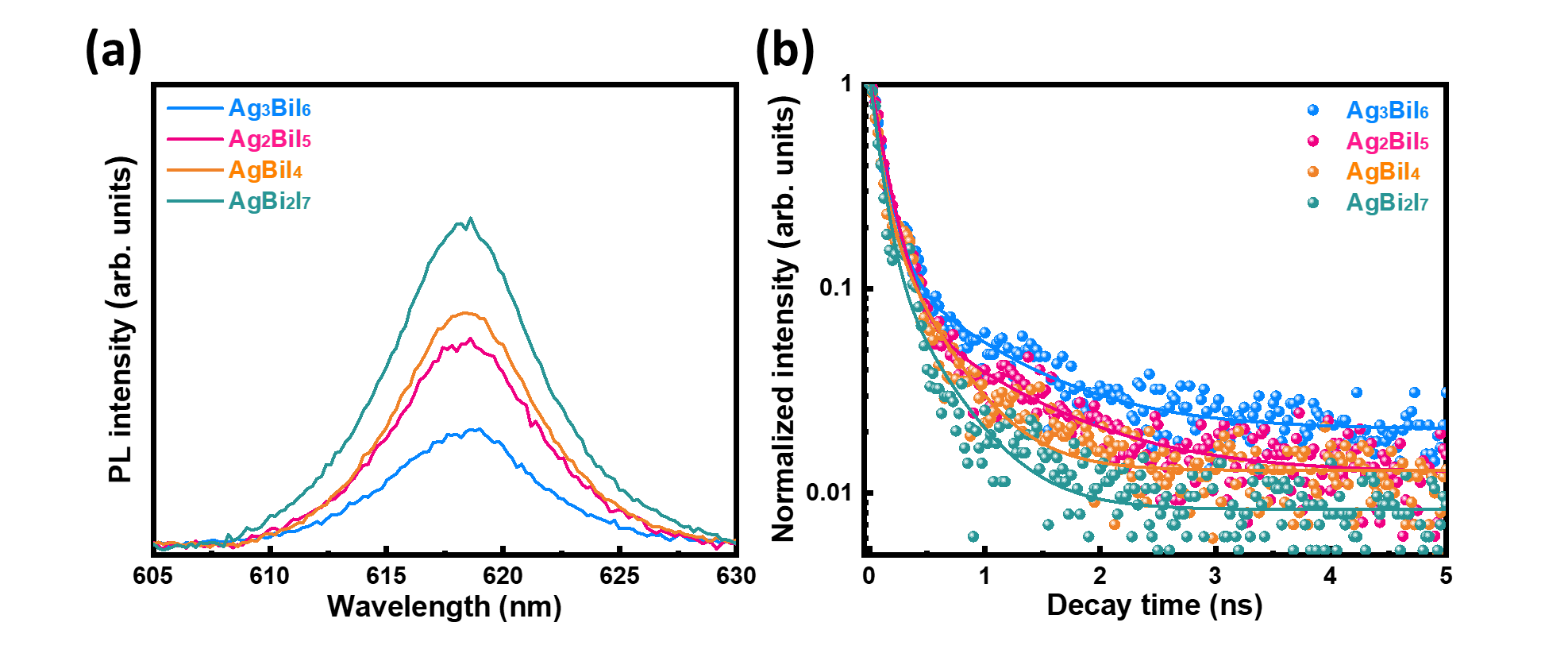


**Figure S15.** Carrier dynamic analysis: **(a)** Steady-state PL spectra and **(b)** TR-PL spectra of various SBI catalysts.


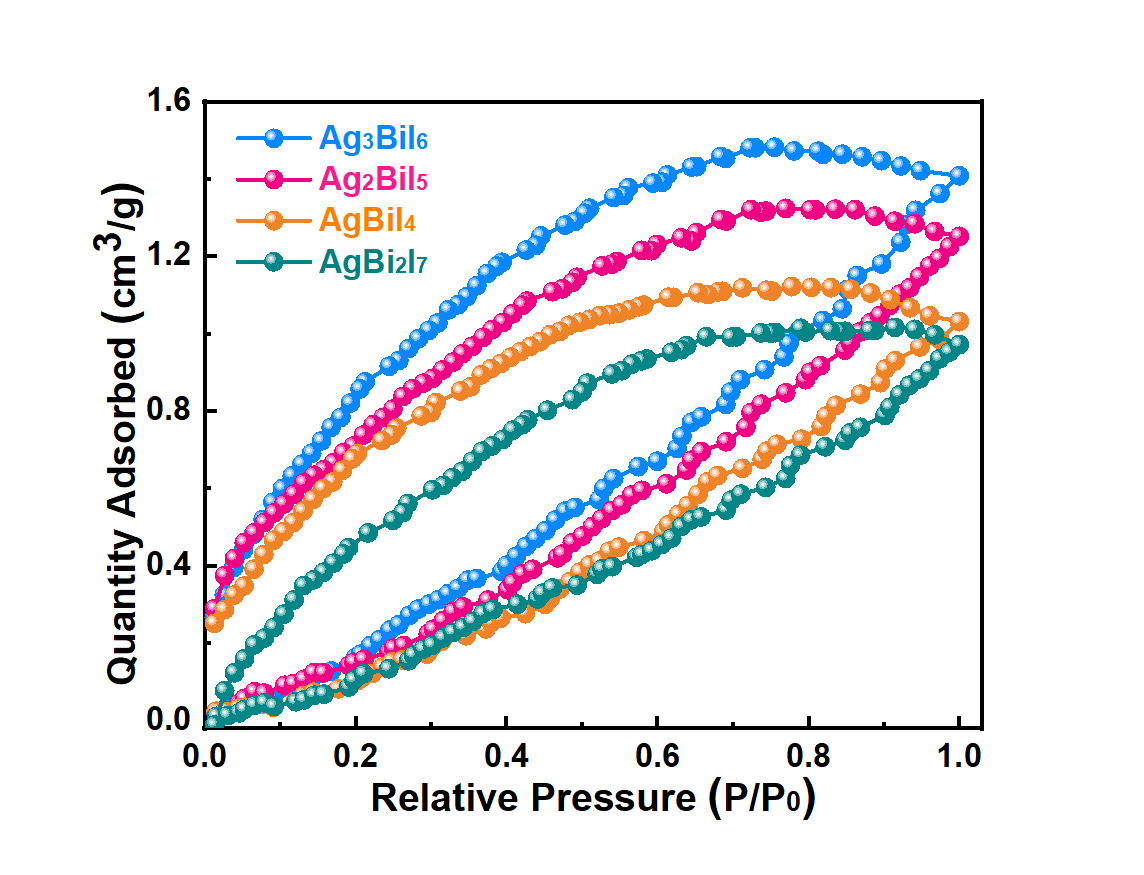


**Figure S16.** CO_2_ adsorption/desorption isotherms of SBI catalysts.


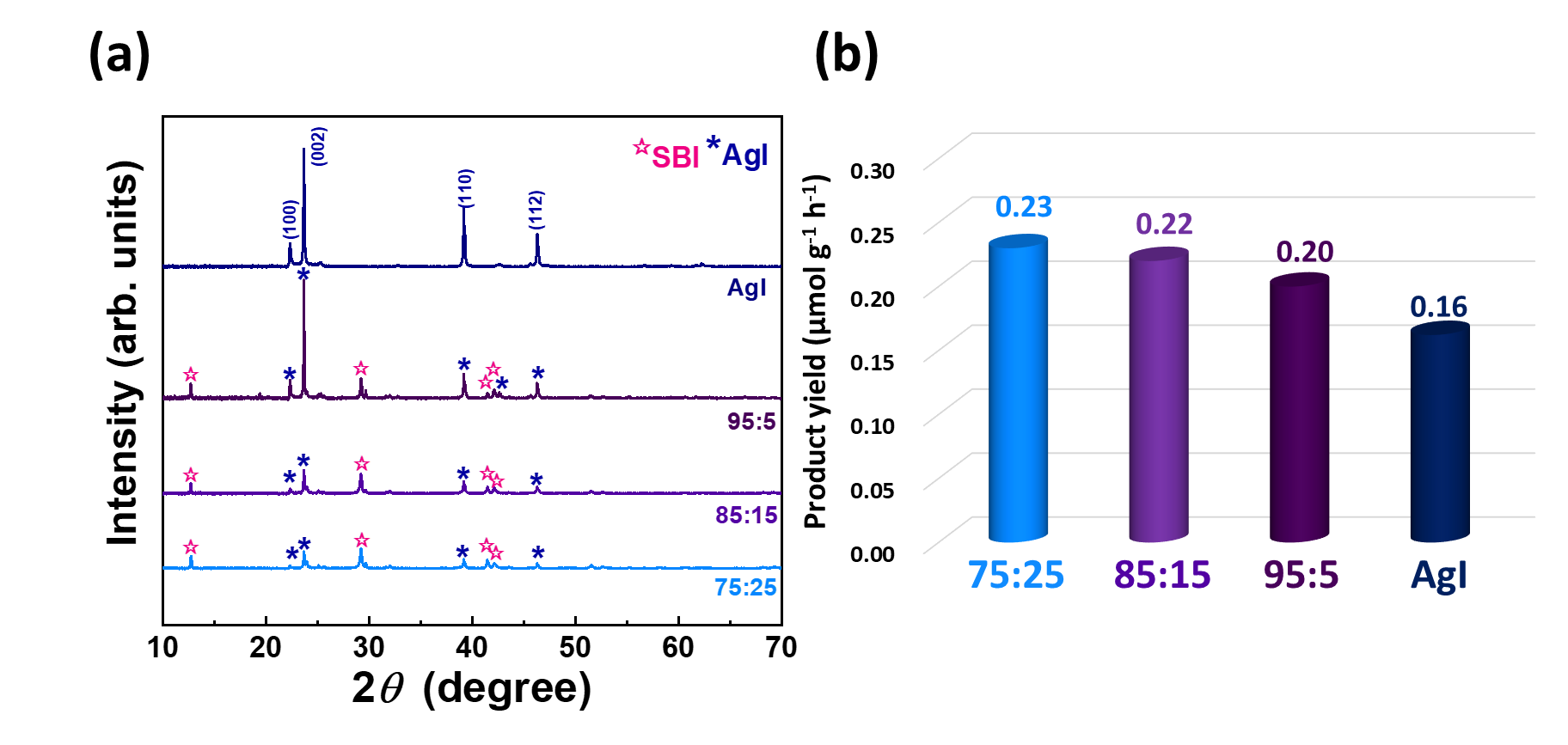


**Figure S17.** **(a)** XRD patterns and **(b)** photocatalytic CO_2_ reduction activities of the CO production rate for AgI and SBI catalysts with AgI to BiI_3_ proportions of 75:25, 85:15, and 95:5.


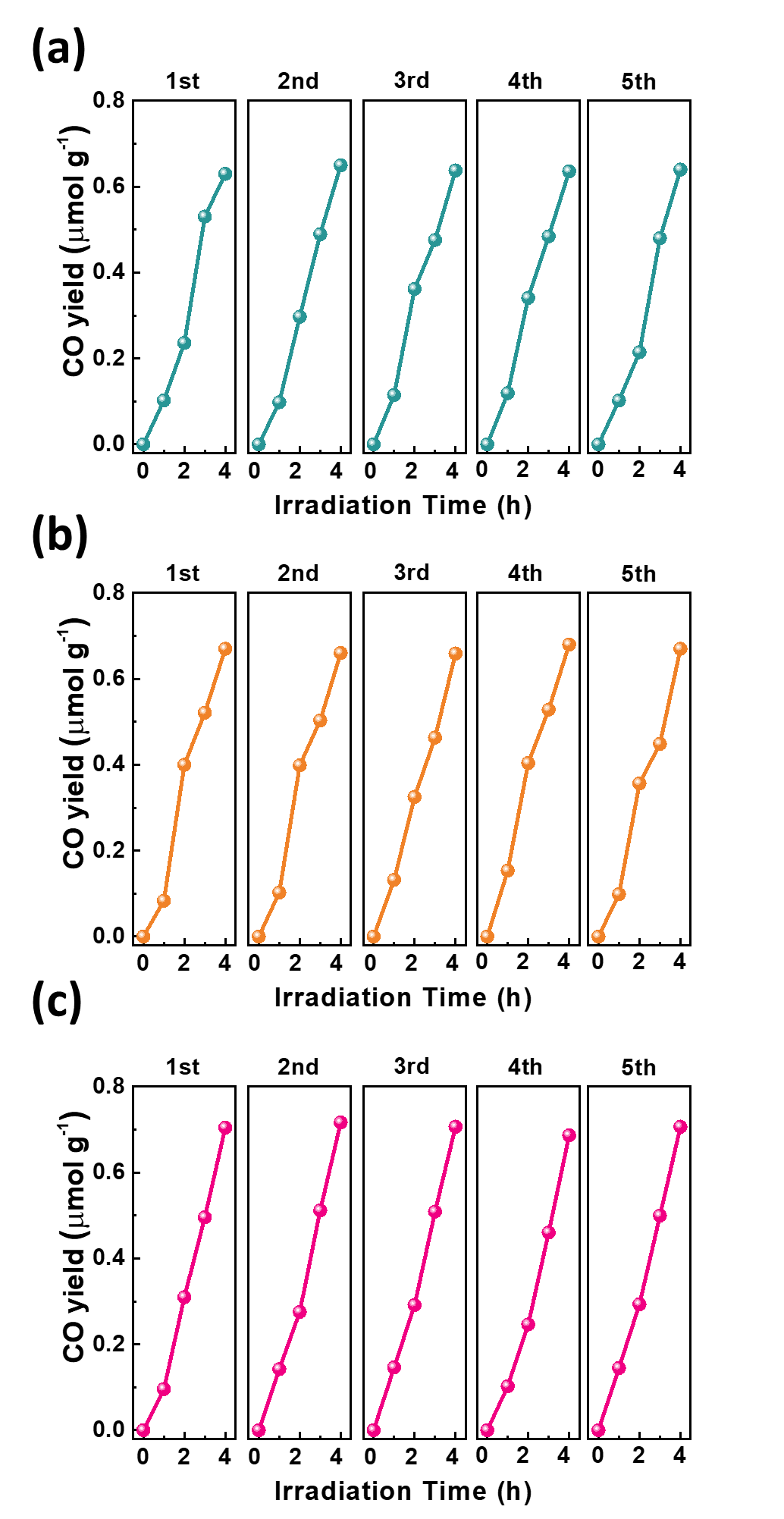


**Figure S18.** Recycling experiments of **(a)** AgBi_2_I_7_, **(b)** AgBiI_4_, and **(c)** Ag_2_BiI_5_.


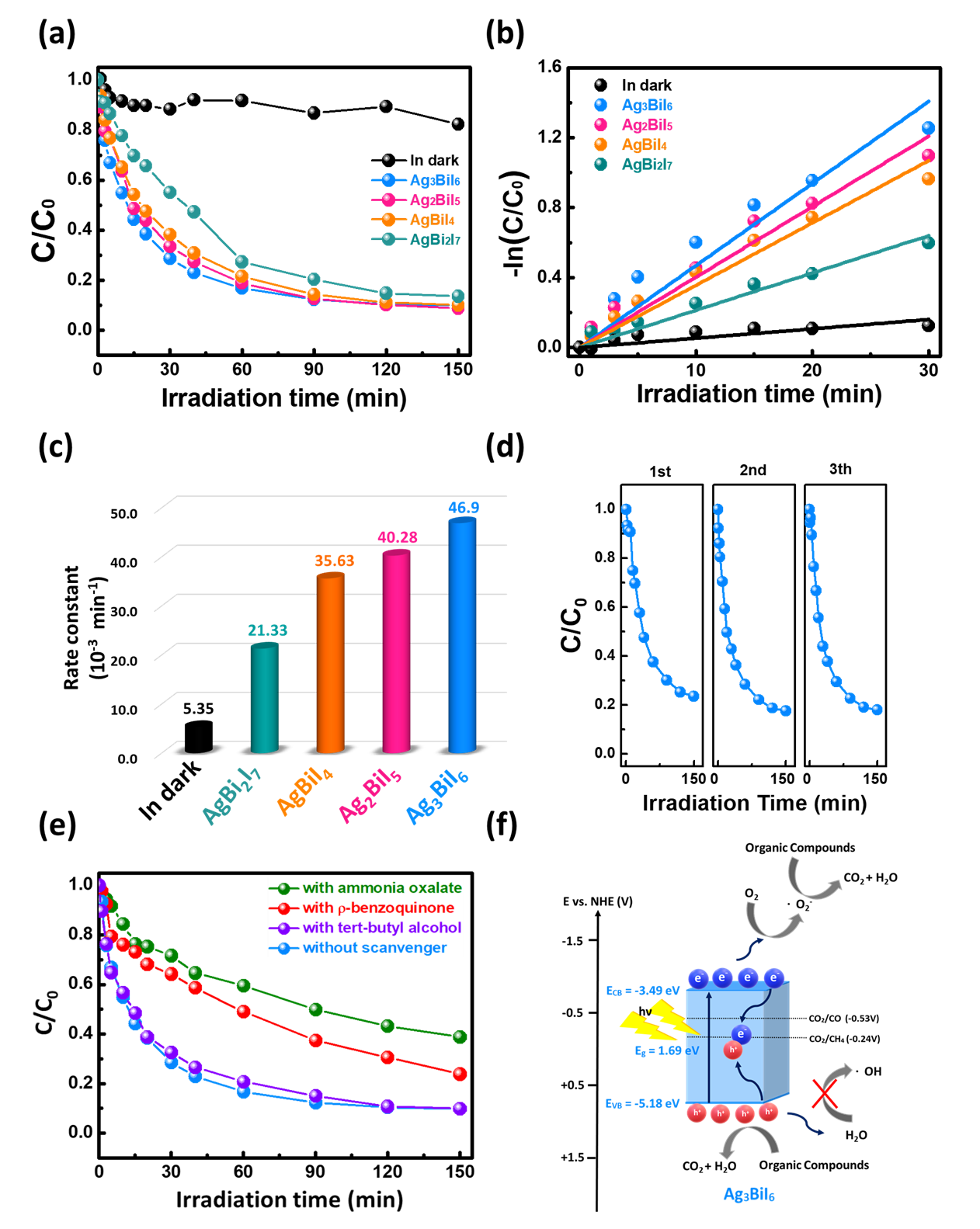


**Figure S19.** Photocatalytic activity analysis of various SBI catalysts: **(a)** photodegradation of brilliant green, **(b)** first-order linear transforms plot of *C/C_0_* curve, **(c)** calculated degradation rate, and **(d)** recycling experiments of Ag_3_BiI_6_. **(e)** Photodegradation curves of brilliant green from Ag_3_BiI_6_ with presence of different scavengers, and **(f)** the proposed photodegradation mechanism.


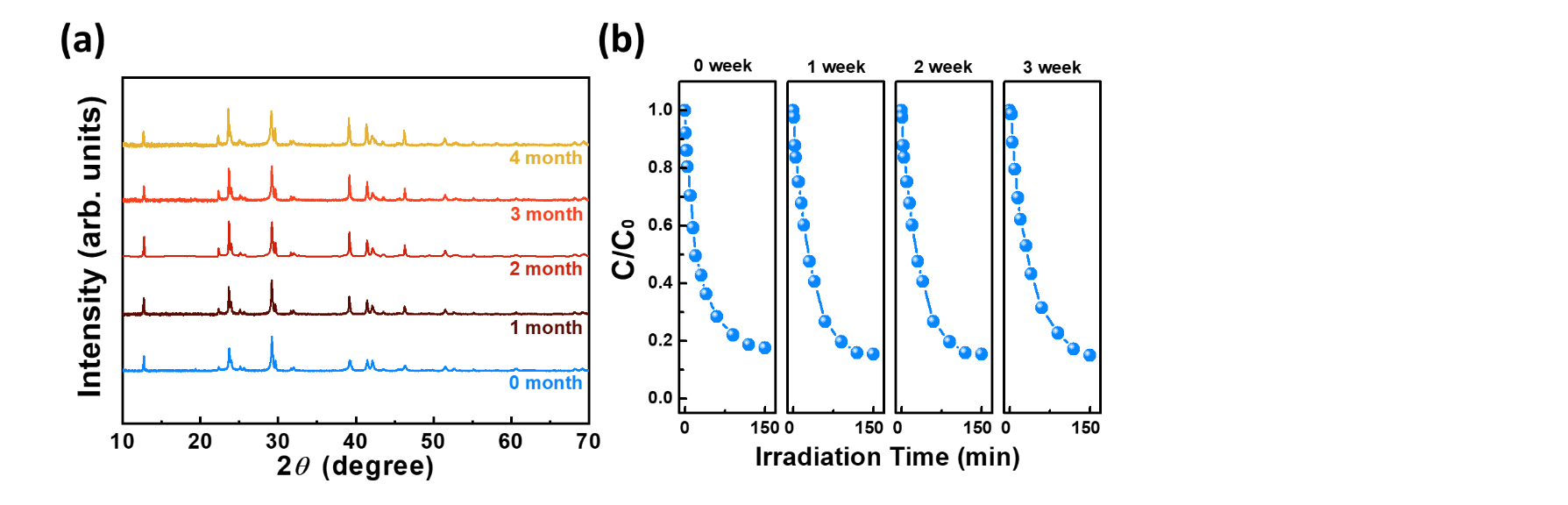


**Figure S20.** Long-term stability test for Ag_3_BiI_6_ photocatalyst: **(a)** XRD pattern, **(b)** photodegradation test of brilliant green.


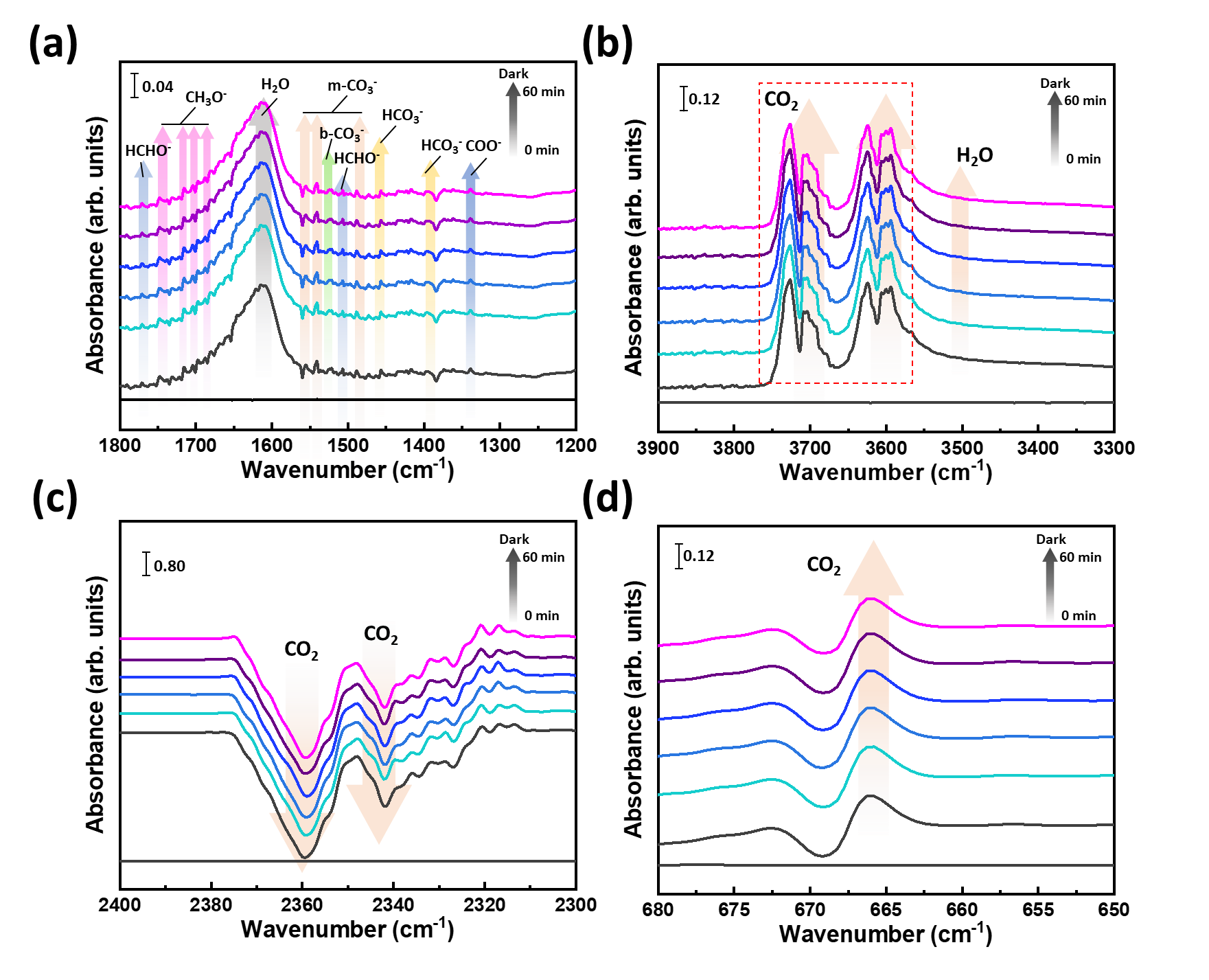


**Figure S21.** In situ DRIFTS spectra of CO_2_ and H_2_O interaction with Ag_3_BiI_6_: **(a)** at 1200-1800 cm^-1^, **(b)** 3300-3900 cm^-1^, **(c)** 2300-2400 cm^-1^, and **(d)** 650-680 cm^-1^ in the dark.

**
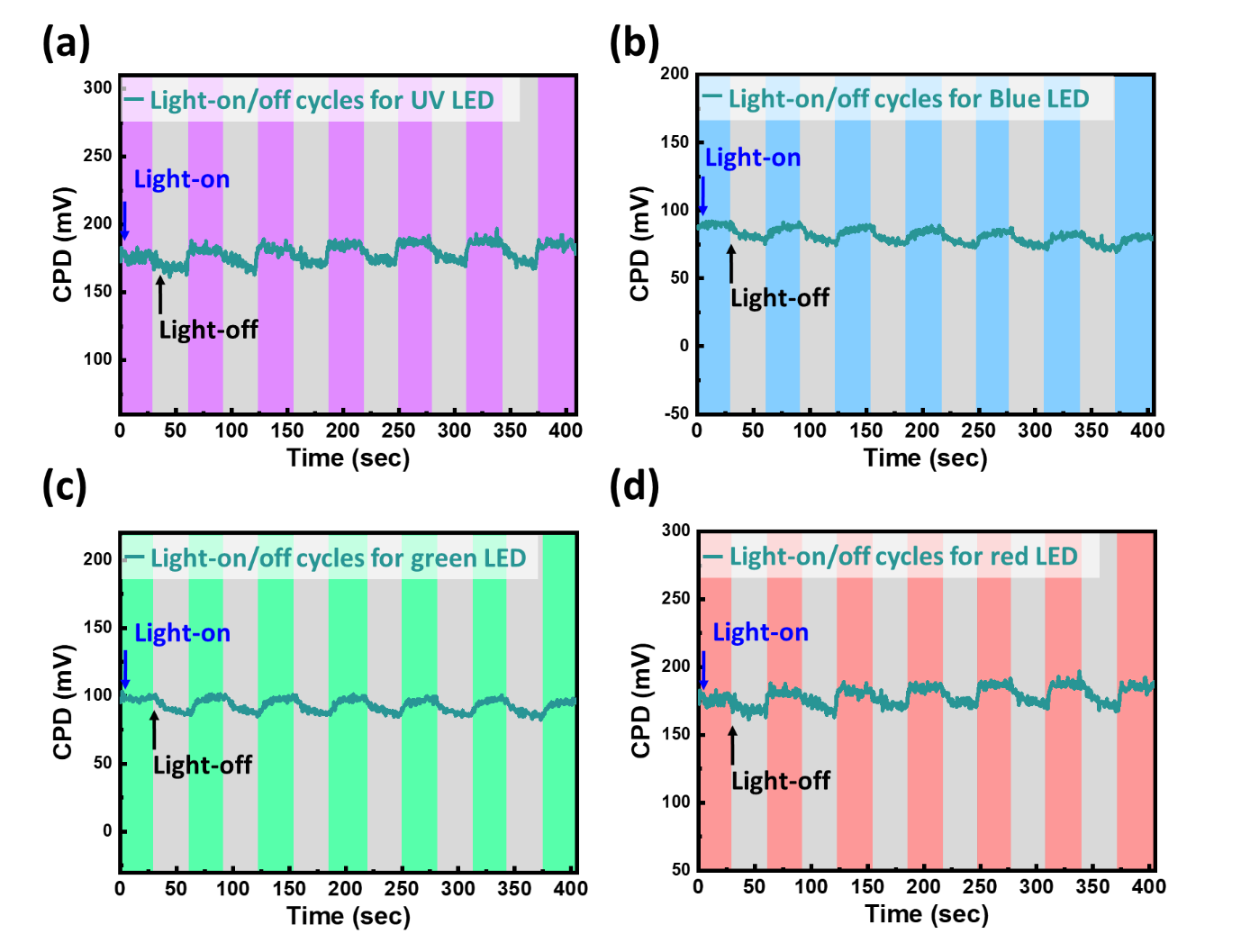
**

**Figure S22.** Contact potential difference with light-on/off cycles for AgBi_2_I_7_ photocatalyst under different LED illumination. **(a)** UV LED (365 nm), **(b)** Blue LED (470 nm), **(c)** Green (530 nm), and **(d)** Red LED (656 nm).

**
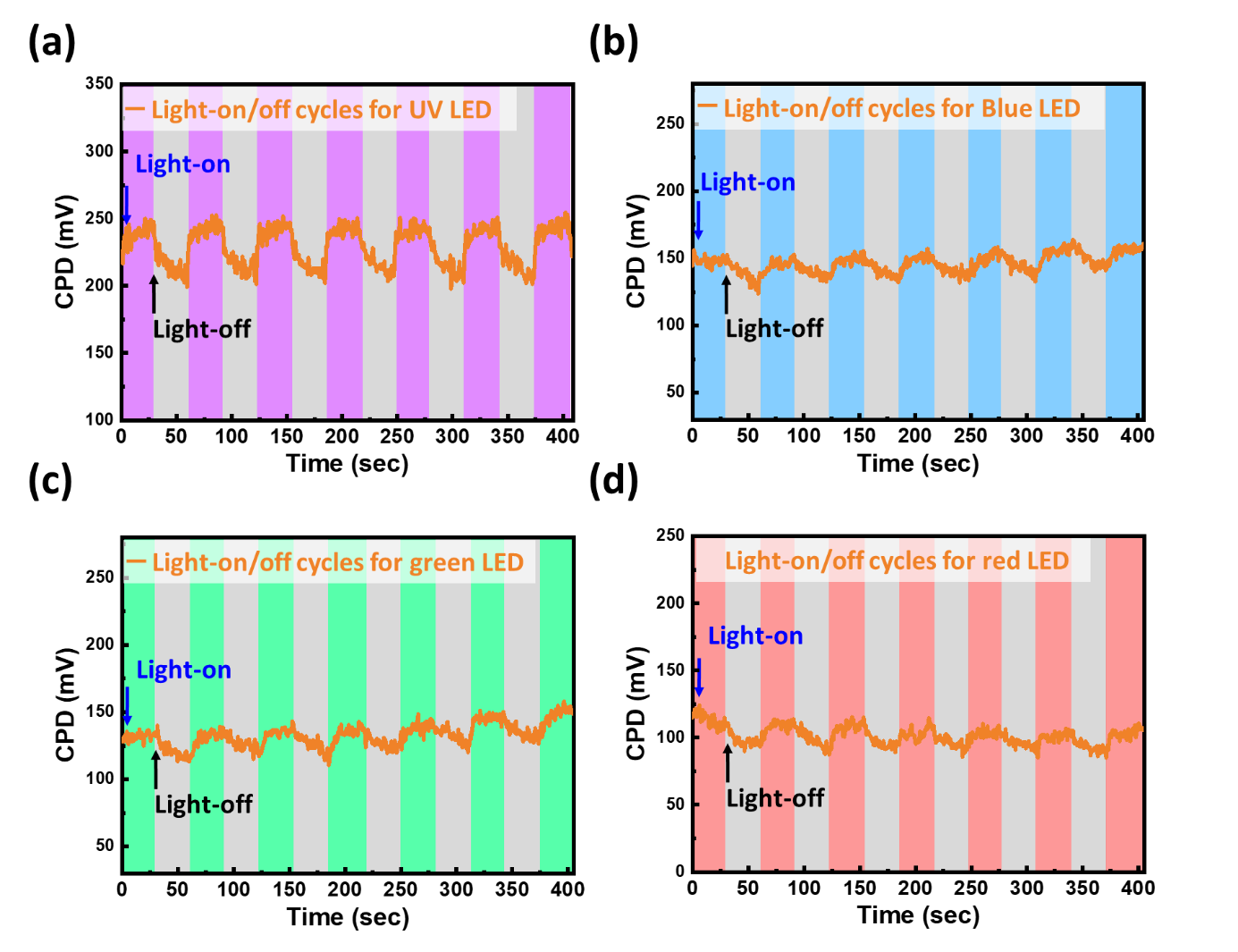
**

**Figure S23.** Contact potential difference with light-on/off cycles for AgBiI_4_ photocatalyst under different LED illumination. **(a)** UV LED (365 nm), **(b)** Blue LED (470 nm), **(c)** Green (530 nm), and **(d)** Red LED (656 nm).

**
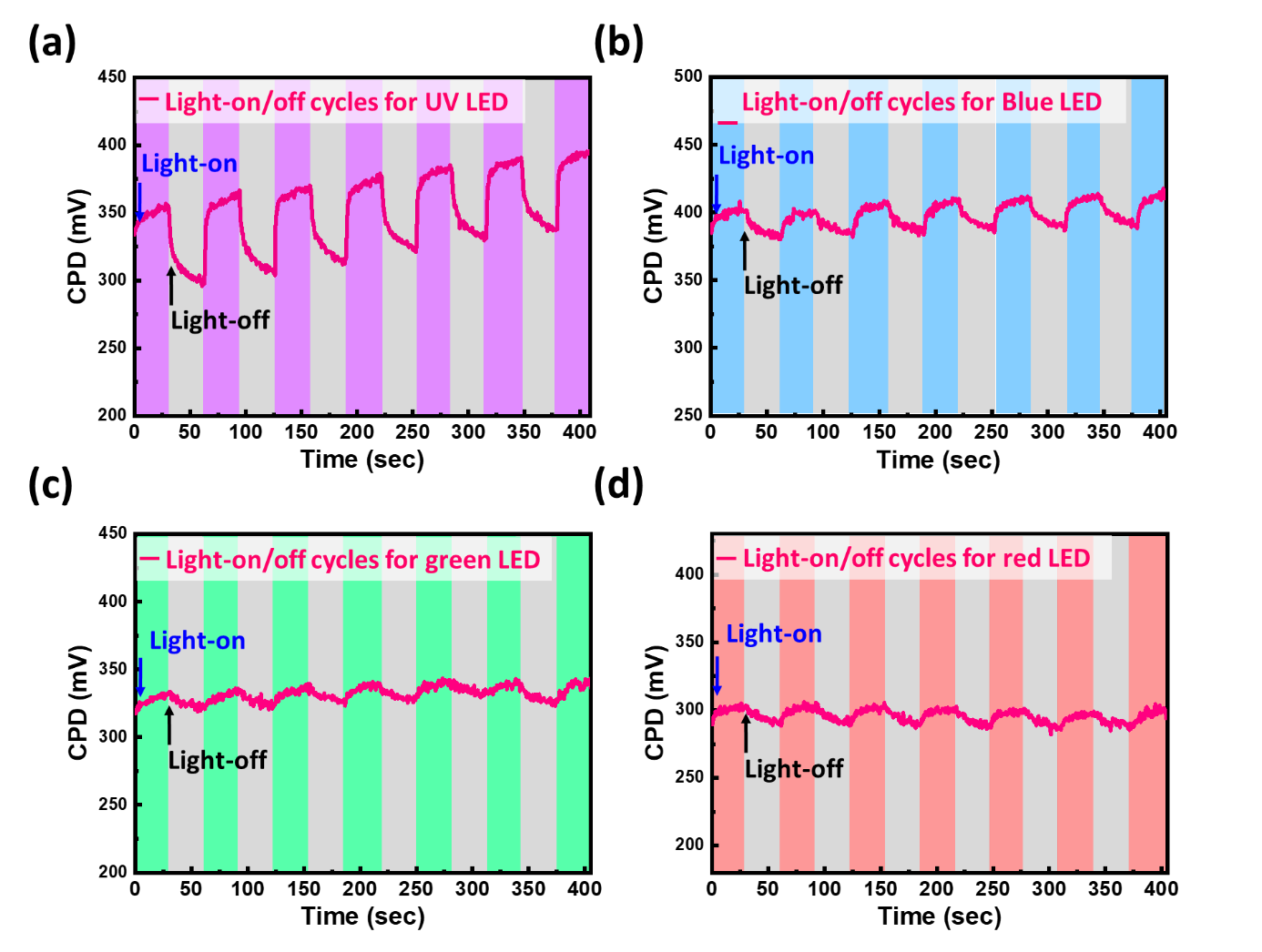
**

**Figure S24.** Contact potential difference with light-on/off cycles for Ag_2_BiI_5_ photocatalyst under different LED illumination. **(a)** UV LED (365 nm), **(b)** Blue LED (470 nm), **(c)** Green (530 nm), and **(d)** Red LED (656 nm).

**
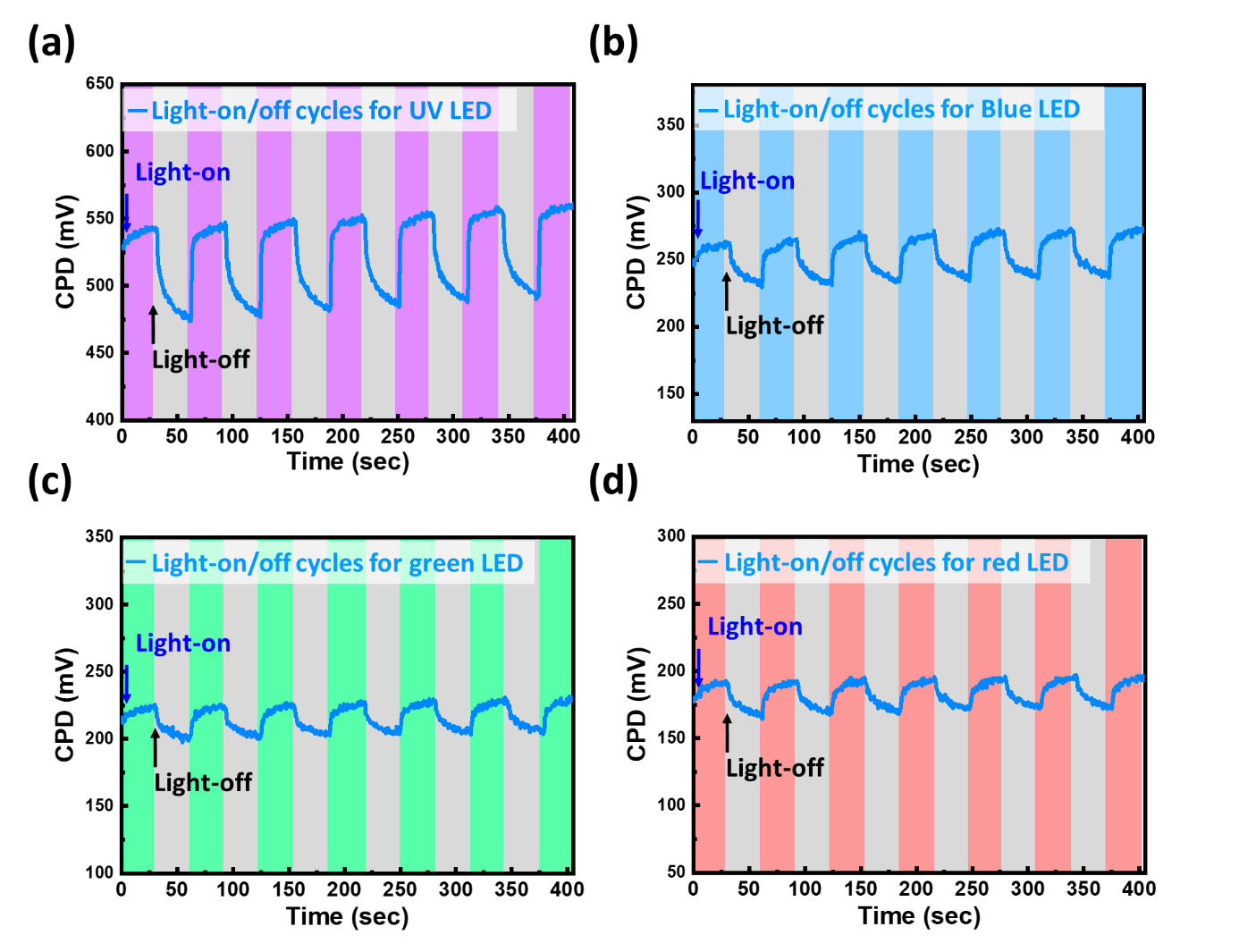
**

**Figure S25.** Contact potential difference with light-on/off cycles for Ag_3_BiI_6_ photocatalyst under different LED illumination. **(a)** UV LED (365 nm), **(b)** Blue LED (470 nm), **(c)** Green (530 nm), and **(d)** Red LED (656 nm).

**
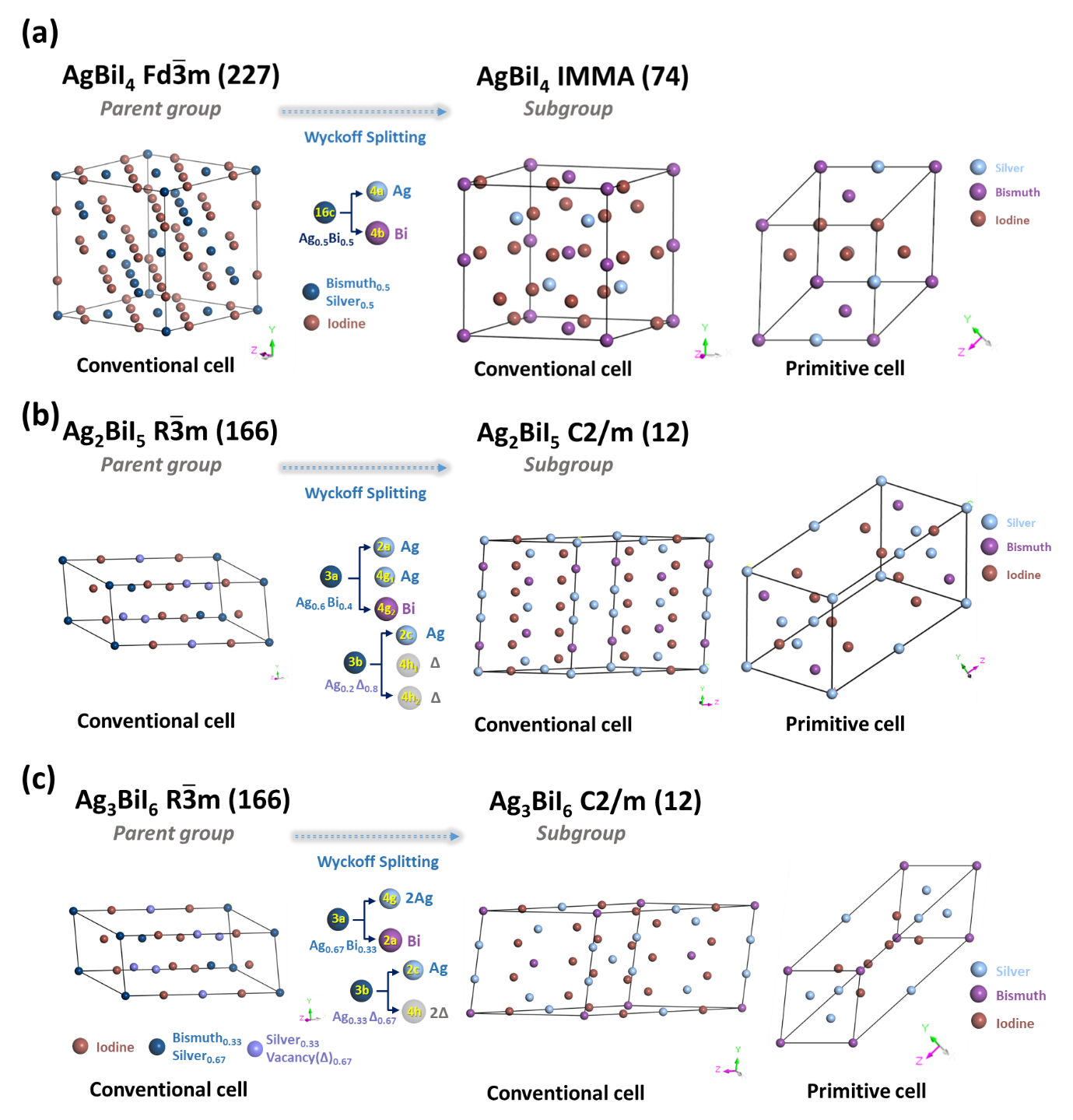
**

**Figure S26**. Model construction of various compounds using the predicted wyckoff position splitting method. **(a)** AgBiI_4_, **(b)** Ag_2_BiI_5_, and **(c)** Ag_3_BiI_6_, illustrating the transformation from the high-symmetry parent group to the low-symmetry subgroup.

Due to the complicated atom sites of silver, bismuth, ioidine and vacant, the wyckoff position splitting approach was employed to provide the reliable model of SBI catalyst.^[S1]^ We also used the Bilbao crystallographic server to realize the wyckoff position splitting.^[ S2- S4]^ Utilizing these method, the specific M matrix links the basis vectors of the parent space group P and transforms it into the subgroup S, denoted as S = PM. Additionally, let m represent the translation matrix that converts the origin p of the parent space group into s of the subgroup, expressed as s = pm.

The pair of matrices M and m that connects the Fd$\bar{\text{3}}$m (227) to Imma (74) for AgBiI_4_: ^[ S1]^

$$\left[ \begin{matrix} \text{1/2} & \text{1/2} & \text{0} \\ \text{-1/2} & \text{1/2} & \text{0} \\ \text{0} & \text{0} & \text{1} \end{matrix} \right]\left[ \begin{matrix} \text{1/4} \\ \text{1/4} \\ \text{0} \end{matrix} \right]$$

The pair of matrices M and m that connects the R$\bar{\text{3}}$m (166) to c2/m (12) for Ag_2_BiI_5_:

$$\left[ \begin{matrix} \text{-1/3} & \text{-5} & \text{0} \\ \text{1/3} & \text{-5} & \text{0} \\ \text{-2/3} & \text{0} & \text{1} \end{matrix} \right]\left[ \begin{matrix} \text{0} \\ \text{0} \\ \text{0} \end{matrix} \right]$$

The pair of matrices M and m that connects the R$\bar{\text{3}}$m (166) to c2/m (12) for Ag_3_BiI_6_: ^[ S1]^

$$\left[ \begin{matrix} \text{1/3} & \text{-3} & \text{0} \\ \text{-1/3} & \text{-3} & \text{0} \\ \text{2/3} & \text{0} & \text{-1} \end{matrix} \right]\left[ \begin{aligned} \text{0} \\ \text{0} \\ \text{0} \end{aligned} \right]$$

**Table S1.** The summarized atomic ratio of Ag^0^, Ag^+^, and Ag^2+^ in Ag 3*d* orbital of various SBI catalysts

| **Sample** | **Atomic ratio (%)** | | |
| --- | --- | --- | --- |
|  | **Ag^0^** | **Ag^+^** | **Ag^2+^** |
| **AgBi_2_I_7_** | N.D. | 66.0 | 34.0 |
| **AgBiI_4_** | 12.0 | 65.7 | 22.3 |
| **Ag_2_BiI_5_** | 19.8 | 67.7 | 12.5 |
| **Ag_3_BiI_6_** | 21.7 | 71.7 | 6.6 |

**Table S2.** The summarized atomic ratio of Bi^2+^ and Bi^3+^ in Bi 4*f* orbital of various SBI catalysts

| **Sample** | **Atomic ratio (%)** | |
| --- | --- | --- |
|  | **Bi^2+^** | **Bi^3+^** |
| **AgBi_2_I_7_** | 25.2 | 74.8 |
| **AgBiI_4_** | 14.8 | 85.2 |
| **Ag_2_BiI_5_** | 7.4 | 92.6 |
| **Ag_3_BiI_6_** | 7.3 | 92.7 |

**Table S3.** The summarized atomic ratio of Ag-I and Bi-I in I 3*d* orbital of various SBI catalysts

| **Sample** | **Atomic ratio (%)** | |
| --- | --- | --- |
|  | **Ag-I** | **Bi-I** |
| **AgBi_2_I_7_** | 40.2 | 59.8 |
| **AgBiI_4_** | 45.1 | 54.9 |
| **Ag_2_BiI_5_** | 55.2 | 44.8 |
| **Ag_3_BiI_6_** | 64.2 | 35.8 |

**Table S4.** EXAFS fitting parameters at the Ag K-edge for various SBI catalysts

| Sample | Path | N^a^ | R (Å)^b^ | σ^2^ (10^3^ Å^2^)^c^ | ΔE_0_ (eV)^d^ | R-factor |
| --- | --- | --- | --- | --- | --- | --- |
| AgBi_2_I_7_ | Ag-I | 6.0 ^f^ | 2.91± 0.08 | 19.1± 0.18 | -0.08± 3.6 | 0.012 |
| AgBiI_4_ | Ag-I | 4.0^f^ | 2.89 ± 0.02 | 15.0± 0.00 | 1.63± 0.7 | 0.001 |
|  | Ag-I* | 2.0 ^f^ | 3.09 ± 0.02 | 14.0± 0.00 | -0.27± 2.5 |  |
| Ag_2_BiI_5_ | Ag-I | 4.0^f^ | 2.83 ± 0.03 | 13.0± 0.01 | 1.66± 0.8 | 0.010 |
|  | Ag-I* | 2.0 ^f^ | 3.01± 0.06 | 14.1± 0.01 | -1.9± 5.1 |  |
| Ag_3_BiI_6_ | Ag-I | 4.0^f^ | 2.88 ± 0.02 | 15.2± 0.01 | 2.8± 1.0 | 0.006 |
|  | Ag-I* | 2.0 ^f^ | 2.98 ± 0.02 | 5.2± 0.01 | -27.0± 4.4 |  |

^a^: coordination number.

^b^: bond distance.

^c^: Debye-Waller factor.

^d^: inner potential correction.

^f^: fixed.

**Table S5.** EXAFS fitting parameters at the Bi L3-edge for various SBI catalysts

| Sample | Path | N^a^ | R (Å)^b^ | σ^2^ (10^3^ Å^2^)^c^ | ΔE_0_ (eV)^d^ | R-factor |
| --- | --- | --- | --- | --- | --- | --- |
| AgBi_2_I_7_ | Bi-I | 6^f^ | 2.95 ± 0.05 | 18.4 ± 0.01 | -7.4 ± 4.6 | 0.019 |
| AgBiI_4_ | Bi-I | 4^f^ | 2.97 ± 0.07 | 35.7 ± 0.40 | -7.2 ± 11.5 | 0.018 |
|  | Bi-I* | 2^f^ | 2.94 ± 0.07 | 9.6 ± 0.03 | -7.2 ± 11.5 |  |
| Ag_2_BiI_5_ | Bi-I | 4^f^ | 2.97 ± 0.03 | 33.0 ± 0.08 | -7.4 ± 3.3 | 0.049 |
|  | Bi-I* | 2^f^ | 2.93 ± 0.03 | 11.6 ± 0.01 | -7.4 ± 3.3 |  |
| Ag_3_BiI_6_ | Bi-I | 4^f^ | 2.97 ± 0.07 | 35.4 ± 0.40 | -7.0 ± 4.4 | 0.019 |
|  | Bi-I* | 2^f^ | 2.93 ± 0.07 | 9.9 ± 0.03 | -7.0 ± 4.4 |  |

^a^: coordination number.

^b^: bond distance.

^c^: Debye-Waller factor.

^d^: inner potential correction.

^f^: fixed.

**Table S6.** The characteristics of slow decay time (*τ_1_*), fast decay time (*τ_2_*), and PL average lifetime (*τ_avg_*) for various SBI catalysts

| **Sample** | ***A*_1_ (%)** | ***τ*_1_ (ns)** | ***A_2_* (%)** | ***τ_2_* (ns)** | ***τ_avg_* (ns)** |
| --- | --- | --- | --- | --- | --- |
| **AgBi_2_I_7_** | 86.11 | 0.08 | 13.89 | 0.40 | 0.12 |
| **AgBiI_4_** | 77.14 | 0.08 | 22.86 | 0.38 | 0.15 |
| **Ag_2_BiI_5_** | 92.52 | 0.12 | 7.48 | 0.86 | 0.18 |
| **Ag_3_BiI_6_** | 88.68 | 0.11 | 11.32 | 0.79 | 0.19 |

**Table S7.** Contact potential differences of various SBI catalysts under different light sources

| **Sample** | ***∆*CPD (mV) under LED illumination** | | | |
| --- | --- | --- | --- | --- |
|  | **UV LED**  **(365 nm)** | **Blue LED**  **(470 nm)** | **Green LED**  **(530 nm)** | **Red LED**  **(656 nm)** |
| **AgBi_2_I_7_** | $\text{12.48 ± 2.26}$ | 1$\text{5.18 ± 1.36}$ | 1$\text{4.25 ± 0.79}$ | 1$\text{5.10 ± 2.17}$ |
| **AgBiI_4_** | $\text{39.60 ± 3.80}$ | 1$\text{9.20 ± 2.83}$ | 1$\text{4.36 ± 2.60}$ | 1$\text{0.83 ± 1.45}$ |
| **Ag_2_BiI_5_** | 5$\text{6.35 ± 3.74}$ | 2$\text{2.22 ± 2.24}$ | 1$\text{4.28 ± 2.38}$ | 1$\text{3.42 ± 1.19}$ |
| **Ag_3_BiI_6_** | 6$\text{6.35}\text{ ± 2.80}$ | 3$\text{2.22 ± 1.92}$ | 2$\text{3.55 ± 0.96}$ | 2$\text{2.21 ± 1.17}$ |

**Table S8**. Lattice parameter of AgBiI_4_ in conventional cell and primitive cell.

| **Space group of AgBiI_4_** | **Length (Å)** | | | **Angle (°)** | | |
| --- | --- | --- | --- | --- | --- | --- |
|  | **a** | **b** | **c** | **α** | **β** | **γ** |
| **Fd**$\bar{\text{3}}$**m-**  **conventional cell** | 12.223 | 12.223 | 12.223 | 90.0000 | 90.0000 | 90.0000 |
| **IMMA-conventional cell** | 8.56087 | 8.683957 | 12.57537 | 90.0000 | 90.0000 | 90.0000 |
| **IMMA-primitive cell** | 8.75842 | 8.75842 | 8.75842 | 121.48667 | 120.56168 | 88.23679 |

**Table S9**. Lattice parameter of Ag_2_BiI_5_ in conventional cell and primitive cell.

| **Space group of Ag_2_BiI_5_** | **Length (Å)** | | | **Angle (°)** | | |
| --- | --- | --- | --- | --- | --- | --- |
|  | **a** | **b** | **c** | **α** | **β** | **γ** |
| **R**$\bar{\text{3}}$**m-**  **conventional cell** | 4.35 | 4.35 | 20.82 | 90.0000 | 90.0000 | 120.0000 |
| **C2/m-conventional cell** | 14.460432 | 21.835729 | 21.19538 | 90.0000 | 170.1607 | 90.0000 |
| **C2/m-**  **primitive cell** | 13.094877 | 13.094877 | 21.19538 | 122.95767 | 122.95767 | 112.97199 |

**Table S10.** Lattice parameters of Ag_3_BiI_6_ in conventional cell and primitive cell.

| **Space group of Ag_3_BiI_6_** | **Length (Å)** | | | **Angle (°)** | | |
| --- | --- | --- | --- | --- | --- | --- |
|  | **a** | **b** | **c** | **α** | **β** | **γ** |
| **R**$\bar{\text{3}}$**m-**  **conventional cell** | 4.3537 | 4.3537 | 20.809999 | 90.0000 | 90.0000 | 120.0000 |
| **C2/m-conventional cell** | 14.27422 | 12.894412 | 21.107514 | 90.0000 | 169.94014 | 90.0000 |
| **C2/m-**  **primitive cell** | 9.617943 | 9.617943 | 21.107514 | 136.94121 | 136.94121 | 84.18525 |

1. B. Cucco, L. Pedesseau, C. Katan, J. Even, M. Kepenekian, G. Volonakis, Solar RRL **2022**, 6, 2200718.
2. M. I. Aroyo, A. Kirov, C. Capillas, J. Perez-Mato, H. J. A. C. S. A. F. o. C. Wondratschek, **2006**, 62, 115.
3. M. I. Aroyo, J. M. Perez-Mato, D. Orobengoa, E. Tasci, G. de la Flor, A. J. B. C. C. Kirov, **2011**, 43, 183.
4. M. I. Aroyo, J. M. Perez-Mato, C. Capillas, E. Kroumova, S. Ivantchev, G. Madariaga, A. Kirov, H. J. Z. f. K.-C. M. Wondratschek, **2006**, 221, 15.
